# The role of short RNA loops in recognition of a single-hairpin exon derived from a mammalian-wide interspersed repeat

**DOI:** 10.1080/15476286.2015.1017207

**Published:** 2015-03-31

**Authors:** Jana Kralovicova, Alpa Patel, Mark Searle, Igor Vorechovsky

**Affiliations:** 1 University of Southampton; Faculty of Medicine; Southampton, UK; 2 Institute of Neuroimmunology; Slovak Academy of Sciences; Bratislava, Slovak Republic; 3 University of Nottingham; Centre for Biomolecular Sciences; School of Chemistry; Nottingham, UK

**Keywords:** bulge, exon, hairpin, mutation, splicing enhancers and silencers, tetraloop, transposable elements, triloop

## Abstract

Splice-site selection is controlled by secondary structure through sequestration or approximation of splicing signals in primary transcripts but the exact role of even the simplest and most prevalent structural motifs in exon recognition remains poorly understood. Here we took advantage of a single-hairpin exon that was activated in a mammalian-wide interspersed repeat (MIR) by a mutation stabilizing a terminal triloop, with splice sites positioned close to each other in a lower stem of the hairpin. We first show that the MIR exon inclusion in mRNA correlated inversely with hairpin stabilities. Employing a systematic manipulation of unpaired regions without altering splice-site configuration, we demonstrate a high correlation between exon inclusion of terminal tri- and tetraloop mutants and matching tri-/tetramers in splicing silencers/enhancers. Loop-specific exon inclusion levels and enhancer/silencer associations were preserved across primate cell lines, in 4 hybrid transcripts and also in the context of a distinct stem, but only if its loop-closing base pairs were shared with the MIR hairpin. Unlike terminal loops, splicing activities of internal loop mutants were predicted by their intramolecular Watson-Crick interactions with the antiparallel strand of the MIR hairpin rather than by frequencies of corresponding trinucleotides in splicing silencers/enhancers. We also show that splicing outcome of oligonucleotides targeting the MIR exon depend on the identity of the triloop adjacent to their antisense target. Finally, we identify proteins regulating MIR exon recognition and reveal a distinct requirement of adjacent exons for C-terminal extensions of Tra2α and Tra2β RNA recognition motifs.

## Abbreviations

TE: transposable element
SINE: Short INterspersed Element
MIR: mammalian-wide interspersed repeat
SSO: splice-switching oligonucleotides
*FGB*: gene for fibrinogen
ESE: exonic splicing enhancer, ESS, exonic splicing silencer.

## Introduction

Introns are removed from eukaryotic genes by a large and dynamic RNA-protein complex termed the spliceosome.^1^ Spliceosomes identify splice sites in mRNA precursors (pre-mRNAs) with single-nucleotide precision and often in a cell type-, developmental stage- or gender-specific manner, contributing to transcriptomic and proteomic diversity and organismal complexity through alternative splicing.^1^ Splice site choice is controlled by conserved but degenerate pre-mRNA signals at intron-exon boundaries and also by auxiliary motifs in introns and exons that activate or inhibit splicing, termed enhancers and silencers.^2-9^ These motifs are preferentially located in single-stranded regions of pre-mRNAs and have been under selection in evolution,^10^ however, their structural correlates are largely unknown.

Numerous examples in the literature show that so-called ‘splicing code’, or information required for accurate RNA processing decisions, is influenced by a sequestration or approximation of pre-mRNA splicing signals.^11-23^ For example, alternative splice sites are enriched for conserved RNA secondary structures and GC-content, which may promote looping out of intervening segments.^24-26^ Long-range RNA structures and intramolecular base pairing have been associated with alternative splicing in *Drosophila* ^27^ and cDNA segments ultraconserved between rodents and humans were found to be AT-rich and resistant to folding.^28^ However, no universal exon recognition rules have been uncovered even for the most common RNA building blocks.

Transposable elements (TEs) are repetitive sequences that can move or transpose themselves to new positions within the genome of a single cell.^29,30^ They constitute up to 80% of eukaryotic genomes, substantially shaping genome evolution, species divergence, exon-intron structure and gene expression.^29,30^ Although each TE family has contributed to protein diversity and can be ‘exonized’, Short INterspersed Elements (SINEs) have been particularly active in evolution in this respect.^31-35^ The overrepresentation of SINEs among exonized TEs was found also for mutation-induced cryptic exons that led to genetic disease.^36^ SINEs occupy ∼13% of the human genome and comprise primate-specific *Alu*s and more ancient mammalian-wide interspersed repeats (MIRs). Each SINE subfamily contributed both traditional^34-38^ and auxiliary^3,34,39-41^ splicing motifs to exonized sequences, thus enhancing proteomic diversity and gene expression control through alternative splicing, but molecular pathways leading to their exonization are only partially understood.

SINEs are overrepresented in introns where they are often present in the opposite orientation within a short distance from each other, which can enhance formation of very stable stem-loop structures and influence RNA processing.^34,42^ Stem-loops (or hairpins) are the most common RNA secondary structure motifs that play an important role in transcription, RNA processing and mRNA export, mRNA stability, subcellular localization, translation and viral replication (reviewed in ref.^43^). Well defined hairpins, such as an iron responsive element (IRE), have been implicated in important cellular functions, including ion uptake and storage, energy metabolism, hypoxic regulation, cytoskeletal and neuronal organization.^43-45^ Highly predictable hairpin structures formed by inverted SINEs may thus provide useful models for studying their importance in splice-site recognition. However, TEs have rarely been in the spotlight of structural models of pre-mRNA splicing, which were focused largely on traditional rather than auxiliary splicing signals.^20^

Here, we have developed a new structural splicing model, consisting of a single-hairpin MIR exon activated by A > G mutation in intron 1 of the human gene for fibrinogen (*FGB*). Using a combination of biochemical and biophysical methods, we show an unexpected autonomy of an IRE-like MIR hairpin in exon recognition and its long-range interaction with an upstream, Tra2-induced exon. Terminal tri- and tetra-loops of the MIR hairpin were sufficient to maintain this autonomy even in the context of a distinct stem but only if coupled with loop-closing base pairs of the original MIR hairpin. We also identify splicing enhancer and silencer categories that best predicted exon inclusion of terminal tri- and tetra-loop mutants. In contrast to terminal loops, splicing activities of internal triloops of the MIR hairpin were predicted by their intramolecular interactions with the antiparallel strand of the helix rather than frequencies of matching trinucleotides in splicing enhancers/silencers. Finally, we identify proteins that control usage of the MIR exon.

## Results

### Splicing activity and stability of a single-hairpin exon

**Figure 1.**
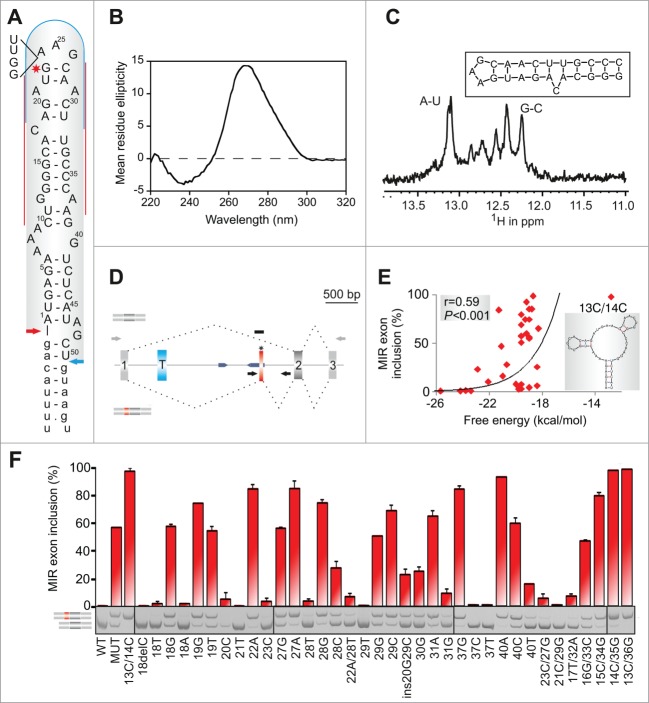
MIR hairpin stabilization is associated with increased exon skipping. (**A**) Predicted RNA secondary structure of the MIR exon. The 5′ and 3′ splice sites are shown as blue and red arrows, respectively. Intronic sequences are in lower case, exon positions (upper case) are numbered. A > G mutation is shown by a star. Alignments of *FGB* intron 1 MIR elements with the MIR consensus is in **Figure S1**; 4-bp sequence deleted in the antisense MIR copy is shown to the left. Putative branch points are indicated in **Figure S1**. Target sequences of splice-switching oligoribonucleotides (SSOs) are shown as red lines (5′ and 3′ stem) and a blue curve (loop SSO). (**B**) Far UV-CD spectrum of the 24-mer hairpin at 25°C showing a strong positive ellipticity at 265 nm. (**C**) 800 MHz ^1^H NMR spectrum of the RNA oligo (*inset*) showing signals for Watson-Crick hydrogen bonded A-U and G-C base pairs between 12–13.5 ppm. (**D**) *FGB* minigene reporter. Exons are shown as boxes, introns as lines; their scale is at the top in base pairs. The MIR and T exons are shown as red and blue boxes; the MIR exon-activating mutation is denoted by a star. Dotted lines show canonical (top) and aberrant (bottom) RNA products. MIRs are denoted by blue arrowheads, cloning primers by black arrows and amplification primers by gray arrows. Primer sequences are shown in **Table S3**. A *FGB* segment cloned into a hybrid *in vitro* splicing reporter (**Fig. 8A**) is denoted by a black rectangle. (**E**) Stabilization of the MIR hairpin increases exon skipping. Free energies were computed for the hairpin shown in panel **A**. A predicted structure of a hairpin destabilising double mutant 13C/14C, which led to full exon inclusion, is shown in the *inset*. (**F**) MIR exon inclusion levels for 38 *FGB* mutations (numbered as in panel **A**). WT, wildtype minigene; MUT, minigene carrying the disease-causing A > G substitution. All mutations were introduced in the MUT minigene; the same mutations in the WT reporter failed to activate the exon (data not shown). Spliced RNA products (*bottom left*) are shown as minigene exons throughout; their color corresponds to that in (**D**). Error bars are SDs of duplicate experiments. Gel panel was merged from separate gels as indicated; cryptic splice sites within or adjacent to the MIR exon were not observed for any construct.

To develop a structural model of auxiliary splicing sequences, we searched previously published cases of exonized TEs in genetic disease^36^ for mutations within SINEs. RNA secondary structure predictions with multiple pre-mRNA segments overlapping new SINE exons revealed that an exonized antisense MIR in *FGB* intron 1 was fully encompassed in a very stable hairpin structure (**Fig. 1A**). As compared to the MIR consensus sequence, this MIR copy sustained a 4-nt deletion that created a purine-rich exonic splicing enhancer (ESE), which was further optimized by the disease-causing A > G mutation (**Fig. S1**), leading to afibrinogenemia.^46^ In this structure, the 5′ and 3′ splice sites of the new exon were positioned close to each other in a stable lower stem whereas the mutation stabilized a closing base-pair of a terminal triloop (**Fig. 1A**).

To support formation of this structure experimentally and test if its stability correlates with inclusion of the MIR exon in mature transcripts, we carried out biophysical studies using a synthetic, MIR-derived RNA (**Fig. 1B, C**) and mutagenesis with minigene constructs containing *FGB* exons 1–3 (**Fig. 1D**). The far-UV circular dichroism (CD) spectrum of a 3 μM buffered solution (pH 7.0) of the 24-mer representing exon positions 13–36 gave a strong positive ellipticity at 265 nm (**Fig. 1B**), consistent with a double-stranded stem-loop structure in solution. The NMR spectrum at 800 MHz of the same oligonucleotide sample showed imino proton signals in the region 12 to 13.5 ppm (**Fig. 1C**), confirming the formation of stable Watson-Crick hydrogen bonded A-U and G-C base pairs. Structure prediction calculations suggested that the oligo hairpin structure (**Fig. 1C**) has a high stability (ΔG = −6.6 kcal mol^−1^) which we examined using CD melting studies. We observed a clear, but broad, melting profile (consistent with mis-matched or unpaired bases) with a mid-point unfolding transition corresponding to a T_m_ = 74.0 ± 3.5°C (data not shown).

Mutagenesis of single- and double-stranded regions of minigene reporters transfected into human embryonic kidney (HEK) 293 cells showed that substitutions of unpaired nucleotides altered exon inclusion levels more than mutations of paired residues (median difference 54.5% *vs* 31.6%, *P* < 0.05) and that inclusion of the MIR exon in the mRNA correlated positively with predicted free energy of mutated constructs (**Fig. 1E, F**). Most notably, the MIR exon inclusion was eliminated by deletion or mutation of a cytosine bulge at position 18, which separates a stable lower helix from a more dynamic upper stem of the hairpin. C or G at this position was associated with exon inclusion and A or T with exon skipping, suggesting that ligand interactions are mediated by hydrogen bonding. Exon inclusion was also diminished by mutations of other single-nucleotide bulges at positions 21, 29 and 37.

Collectively, computational, CD, NMR and mutagenesis studies supported the structural model of MIR exon activation shown in **Figure 1A**. This model permits systematic manipulation of terminal loops without altering splice-site configuration of the new exon, thus providing an insight into structural correlates of auxiliary splicing sequences.

### MIR exon inclusion levels of 64 RNA triloops

**Figure 2.**
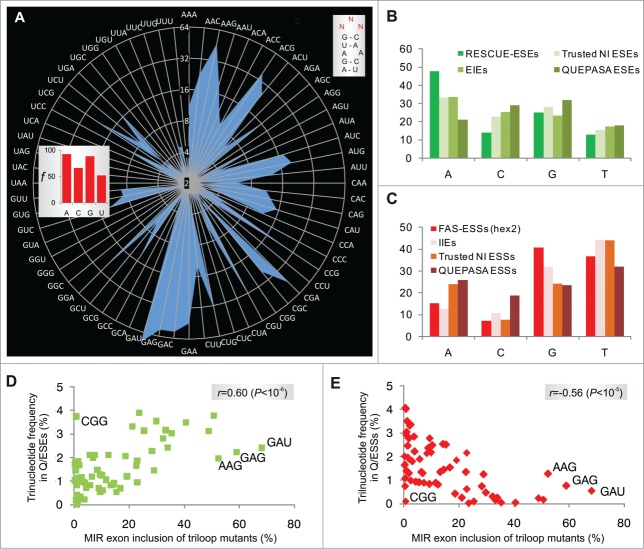
RNA triloops as structural correlates of splicing enhancers and silencers. (**A**) Mean inclusion levels of 64 terminal triloop mutants of the MIR hairpin exon (top right; mutated residues are in red). Inclusion levels are on a *ln* scale. *Inset* shows weighted triloop nucleotide-specific contribution to exon inclusion levels (see Materials and Methods). (**B, C**) Nucleobase fraction (%) of the indicated categories of splicing enhancers and silencers. Splicing enhancers (**B**) and silencers (**C**) were published previously as RESCUE-ESEs,^4^ exon and intron identity elements (EIEs and IIEs),^7^ ESEs and ESSs derived by neighborhood inference (trusted NI ESEs and ESSs) ^77^ and QUEPASA ESEs and ESSs.^9^ (**D, E**) MIR exon inclusion levels of MIR triloop mutants were best predicted by frequencies of corresponding trinucleotides in QUEPASA hexamers.^9^ Pearson correlation coefficients and associated P values are shown at the top. Trinucleotide frequencies in QUEPASA enhancers and silencers are shown in **Figure S3**. Correlation matrix for all enhancer and silencer classes is in **Table S1**.

Next, we cloned 64 minigenes representing all possible nucleotide combinations in the terminal triloop (exon positions 24–26, **Fig. 1A**) and determined inclusion levels of mutated MIR exons after transfection (**Fig. 2A**). The GAN triloops (where N is any base) produced the highest exon inclusion levels, accounting for over a third of cryptic splicing, while the GA-and AG-containing loops contributed >50% to the total. The A > G > C > U hierarchy of triloop nucleotide splicing activities (**Fig. 2A**, *inset*) was the same as base frequencies found for most splicing enhancer categories (**Fig. 2B**). Silencers showed the opposite order (data not shown; cf. **Fig. 2C**). Notably, our triloop mutants exhibited almost identical hierarchy in exon inclusion levels in 4 different primate cell lines although epithelial HeLa cells produced systematically less cryptic splicing than the remaining tested cells (**Fig. S2**).

The most stable secondary structures predicted for each triloop mutant maintained the identical hairpin in 60/64 (94%) cases, altering only the terminal triloop display. The four remaining loop trinucleotides (CCC, GGG, AGG and GGC) and stable alternative secondary structures predicted for CCU, CUC, UCC and UGC triloops lacked the cytosine bulge; remarkably, they all failed to activate the MIR exon (**Fig. 2A**).

Taken together, splice-switching properties of single nucleotide bulges of the hairpin were complemented by a remarkably wide range of splicing activities of terminal triloops, in which GAN/NAG trinucleotides contributed most to exonization. The order of triloop splicing activities was very similar across primate cell lines.

### Trinucleotide frequencies in splicing enhancers and silencers correlate with splicing activities of corresponding terminal triloops

To determine the extent to which each triloop mutant contributes to universal exon recognition, we correlated their exon inclusion levels with trinucleotide frequencies in previously defined categories of splicing enhancers and silencers. A significant positive correlation was found for most enhancer categories and negative correlation for most silencer classes (**Fig. 2D, E**). The highest correlation was found for trinucleotide frequencies in the most recently derived class of enhancers (termed QUEPASA), particularly when normalized for their previously defined ESEseq scores^9^ (**Table S1**, **Fig. 2D**). The pattern of splicing activities of 64 terminal MIR triloops strikingly resembled the radar chart showing frequencies of matching trinucleotides in QUEPASA enhancers (*cf.* **Fig. 2A** and **Fig. S3**); this pattern was dramatically distinct for QUEPASA silencers. Thus, the terminal triloop identity contributed to a great extent to enhancer/silencer activities of the MIR exon.

### Triloop identity controls splicing outcomes of an adjacent antisense target

**Figure 3.**
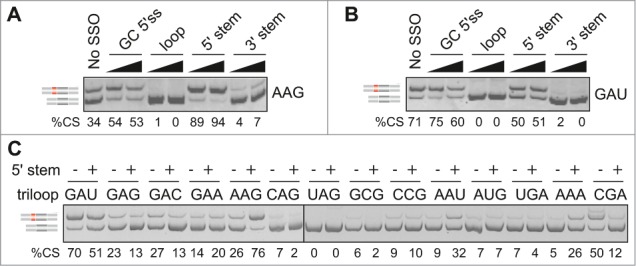
Triloop-dependent promotion and repression of the MIR exon by SSOs. (**A, B**) Identification of SSOs that target the MIR exon and promote its inclusion in mature transcripts. SSOs are shown at the top, percentage of cryptic splicing (%CS) at the bottom, spliced products schematically to the left and tested triloop sequence to the right. SSO sequences are shown in **Table S3** and their hairpin targets in **Fig. 1A**. Final concentrations of each SSO in cell cultures were 40 and 80 nM. GC 5'ss denotes a 2′-*O*-methyl SSO used as one of the controls; this SSO targets the 5′ splice site of a cryptic exon in the *BTK* gene and induces exon skipping ^74^ and is also partially complementary to the 5'stem MIR at exon positions 8–10 and 13–17 (**Fig. 1A**). (**C**) Triloops that promote or repress MIR exon inclusion upon binding of the 5′ stem SSO. A final concentration of the SSO in the culture medium was 50 nM. The panel was merged from 2 separate gels.

To further test the relative importance of single and double-stranded regions in MIR exon recognition, we employed splice-switching oligonucleotides (SSOs) that targeted the 5'stem, terminal loop and 3'stem of the hairpin (**Fig. 1A**). All SSOs were 2'-*O*-methyl-modified at each sugar residue and uniformly labeled with phosphorothioates (**Table S3**). Cotransfection of loop and 3'stem SSOs with *FGB* reporters produced invariably exon skipping. Interestingly, the effect of the 5'stem SSO depended on the loop identity (**Fig. 3A, B**). This SSO promoted exon inclusion of terminal loop mutants that contained diadenosines (except for CAA and AAC) but increased exon skipping or had no effect for the remaining loops (**Fig. 3C**). Thus, the splicing outcome of the SSO that targeted a more accessible face of the MIR hairpin was controlled by the adjacent single-stranded sequence.

### MIR hairpin acts as an autonomous exon selection module

**Figure 4.**
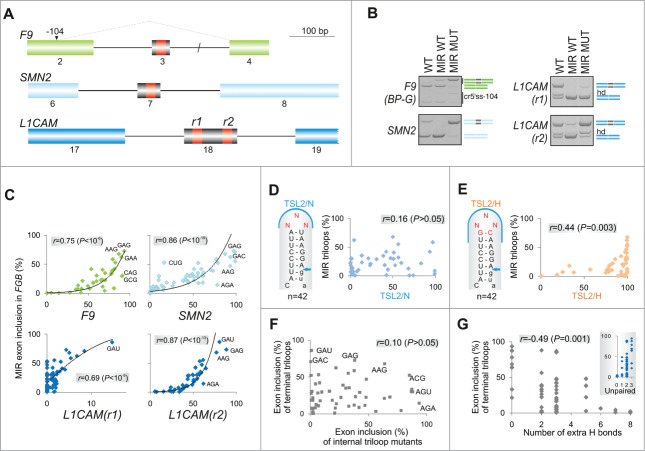
Loop- and stem-dependencies of MIR exon recognition. (**A**) Hybrid reporter constructs. Exons are shown as boxes (numbered and to a scale shown at the top); introns (not to a scale) are shown as lines. Splicing to a cryptic 5′ splice site in *F9* (cr5ss-104) ^73^ is shown by dotted lines. MIR hairpins are in red; r1 and r2 denote their positions in 2 *L1CAM* constructs. (**B**) Spliced products of wild-type (WT) reporters and their hybrid counterparts with (MIR MUT) and without (MIR WT) the A > G transition. hd, heteroduplexed PCR products. *F9* BP-G constructs had substitution of the branch point adenine for guanine ^73^ to obtain sufficient expression of transcripts skipping exon 3. (**C**) Exon inclusion levels (%) of terminal triloop mutants in *FGB* (*y-axes*) and of corresponding triloop mutants in the indicated hybrid pre-mRNAs (*x-axes*). Outliers/purine-rich triloops are highlighted. r, Pearson correlation coefficient. (**D**) Splicing activities of *FGB* MIR and *SMN2* TSL2 triloop mutants. Secondary structure of the native TSL2 hairpin (TSL2/N) ^21,47^ is shown schematically to the left; mutated nucleotides are in red. The 5′ splice site of *SMN2* exon 7 is denoted by an arrow. The number of tested triloops is shown at the bottom. (**E**) Loop-closing base-pairs significantly contribute to the context-dependency of splicing reactions. *x-axis* shows splicing activities of corresponding triloop mutants in *SMN2* TSL2/H. Heptamers shared between MIR (**Fig. 1A**) and hybrid (TSL2/H) triloop mutants are denoted by a blue curve; a shared sequence between TSL2/N and MIR is limited to the triloop, as in panel D. (**F**) A lack of correlation between exon inclusion levels of matching internal and terminal triloop mutants in *FGB*. Triloops generating high exon inclusion are indicated. (**G**) MIR exon inclusion levels of internal triloop mutants inversely correlate with the number of hydrogen bonds formed between mutated triloops and the antiparallel strand. The number of hydrogen bonds (*x-axis*) was calculated from Mfold predictions for each mutant (**Table S2**). *Inset* shows that MIR exon inclusion levels (%, *y-axis*) significantly increased with the number of unpaired bases between the internal loop and the antiparallel strand of the MIR hairpin (*P* <0.001).

To test generality of the observed triloop hierarchies of exon usage (**Fig. 2A**) and the role of their sequence context in exon recognition, we examined splicing activities of the same 64 hairpins inserted in central exons of 3 additional minigenes. MIR hairpins were cloned in *F9* exon 3, *SMN2* exon 7 and at 2 insertion sites of *L1CAM* exon 18 (**Figs. 4A** and **S4**). If exon recognition is facilitated largely by autonomous hairpin interactions through the terminal loop, we should observe a similar order of exon inclusion levels for triloop mutants in *FGB* and hybrid transcripts and a significant correlation of exon inclusion levels between *FGB* and hybrid mutants. In contrast, if the inclusion of central exons relies more on other interactions, we would expect a more variable ranking of their triloop-specific splicing activities.

**Figure 4B** shows that the MIR hairpin containing the disease-causing A > G substitution (MUT) increased inclusion of the central exon in each hybrid minigene. By contrast, the hairpin lacking this substitution and unable to activate the MIR exon (WT) induced exon skipping in *SMN2* and *L1CAM*, nevertheless it could still promote inclusion of a very small exon in the *F9* minigene. We then transfected 256 sequence-verified constructs (i.e., 64 triloops for each hybrid) into HEK293 cells and determined inclusion of central exons in their mRNAs. As shown in **Figure 4C**, exon inclusion of 64 triloop mutants in the context of *FGB* pre-mRNAs positively correlated with exon inclusion of the same triloop mutants in each hybrid minigene. We conclude that the terminal triloop identity of the MIR hairpin was a major determinant of exon inclusion in each hybrid transcript, acting autonomously through its ligand interactions.

### Loop-closing base-pairs as a principal contributor to stem-specific splicing activities

To determine the relative importance of stem identity of this potentially universal exon-promoting structure, we investigated the TSL2 hairpin in *SMN2* exon 7, which terminates with the AAU triloop^21,47^ (**Fig. S4A**). We randomly mutated this triloop in the *SMN2* minigene, measured exon 7 inclusion of the resulting 42 TSL2 triloop variants and correlated their inclusion levels with those established for terminal triloops in the *FGB* minigene (**Fig. 4D**). In the native *SMN2* context (TSL2/N), exon 7 inclusion levels was not significantly correlated with the MIR exon inclusion of *FGB* constructs, suggesting that the triloop identity alone does not predict splicing activity. However, when the A-U closing base pair of TSL2 was replaced by G-C in each mutant, thereby extending the shared motif between *SMN2* and *FGB* hairpin caps to heptamers, the hybrid (TSL2/H) or ‘MIR-onized’ reporters produced significant correlation (**Fig. 4E**).

We next tested the importance of the loop-closing guanine for MIR exon recognition. We exchanged the loop-closing bases in a randomly chosen subset of *FGB* triloop mutants and examined their exon inclusion levels. The GC pair yielded higher exon inclusion than the CG pair in 8 of 10 tested constructs. The splicing enhancement was particularly pronounced in pre-mRNAs in which the 5′ guanine in the base-pair was followed by an unpaired adenine at the first triloop position (**Fig. S5**).

Taken together, these data showed that the stem dependency of MIR exon activation was to a significant extent determined by the identity of loop-closing base-pairs. They also explain previous observations of higher predictive values of hexa- or octamer splicing enhancers for exon inclusion than for shorter oligomers. Finally, the higher exon inclusion of G(NNN)C triloops as compared to their C(NNN)G counterparts (**Fig. S5**) may reflect a previously described lower thermodynamic stability of the former^48^ and contribute to the observed positive correlation between splicing activity of the hairpin and free energy (**Fig. 1E**).

### Intramolecular base-pairing interactions of the internal triloop predict MIR exon inclusion

Apart from the terminal triloop, the MIR hairpin contains a predicted internal triloop (or trinucleotide bulge) located closer to the 3′ splice site at exon positions 7–9 (**Fig. 1A**). Unlike the terminal triloop, however, systematic mutagenesis of the internal triloop would introduce new base-pairing contacts with the antiparallel strand of the hairpin. To explore their significance in MIR splicing, we randomly mutagenized this unpaired segment in the *FGB* reporter, measured exon inclusion levels of the resulting mutants and compared them to the number of predicted hydrogen bonds between triloop mutants and the antiparallel strand of the hairpin (**Table S2**). Interestingly, inclusion levels of internal triloop mutants correlated neither with corresponding terminal loop mutants (**Fig. 4F**) nor with frequencies of matching trinucleotides in splicing silencers and most enhancers (**Table S1**). By contrast, exon inclusion levels of internal triloop mutants significantly correlated with the number of hydrogen bonds between mutated trinucleotide and the antisense sequence (**Fig. 4G**). Together, these data further support a critical role of hairpin structures and their stabilities in exon recognition and suggest that activities of splicing enhancers and silencers, particularly motifs that show the highest correlation with splicing outcomes in our minigenes (**Table S1**, **Figs. 2D, E**), are better predicted by exposed rather than less accessible short RNA loops.

### Triloops frequent in previously determined RNA secondary structures tend to confer low inclusion of the MIR exon

Are inclusion levels of 64 MIR triloop mutants related to triloop frequencies in natural RNAs? To begin to answer this question, we compared frequencies of 823 triloops in previously determined 1349 RNA secondary structures and the MIR exon inclusion of matching triloop mutants. The triloop database was derived from structures of 123 small subunit rRNAs, 223 large subunit rRNAs, 309 5S rRNAs, 484 tRNAs, 91 signal recognition particles, 16 RNase P RNAs, 100 group I introns and 3 group II introns^48^ (Brent Znosko, personal communication). Interestingly, triloops frequent in natural RNAs tended to have low exon inclusion values and *vice versa* (**Fig. S6A**). The only outlier was the GAA triloop: although relatively frequent in nature, it was associated with a high inclusion of the MIR exon, both in *FGB* and hybrid transcripts. In contrast, we observed no significant correlation for the internal MIR loop mutants, in which the GAA triloop outlier was not dominant (**Fig. S6B**). These data suggest that triloops most frequent in nature (including UAA, GCA, UUU and AUA) tend to have splicing silencer activitites, except for GAA, probably the most potent splicing enhancer motif.^36,49^ With the notable exception of GAA, triloops common in these natural RNAs thus appear to contribute more than expected to repression of pseudo-splice sites, which outnumber authentic splice sites by an order of magnitude.^3^ A key assumption of this hypothesis is that the number and type of triloops in the database are representative of triloops formed in naturally occurring pre-mRNAs immediately upon transcription, which remains to be tested.

### RNA tetraloops that promote inclusion of the MIR exon

**Figure 5.**
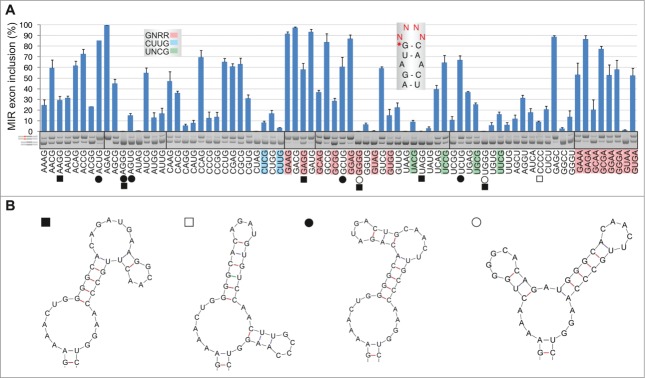
RNA tetraloops that promote and repress MIR exon splicing. (**A**) MIR exon inclusion levels of 76 constructs with the indicated tetraloops in COS7 cells. RNA products are to the left, merged gels are denoted by vertical lines. Error bars represent SDs from 2 transfections experiments. Stable tetraloops are colored as indicated. A default option of Mfold identified 11 transcripts (marked by symbols at the bottom) with stable alternative RNA secondary structures (shown in panel **B**); predicted structure of the remaining pre-mRNAs is shown in the *inset* of panel A.

The most common terminal loops in natural RNAs have 4 nucleotides.^50^ To test their exon-enhancing activities, we converted our terminal triloop mutants into tetraloops, including stable UNCG-, CUUG- and GNRA-type tetraloops that are preferred in nature,^50^ and examined exon inclusion of mutated reporter constructs in the *FGB* mRNA. As with terminal triloops, GA-containing tetraloops gave the highest level of exon inclusion (**Fig. 5A**). The GNRR-type tetraloops tended to be overrepresented among splice-proficient pre-mRNAs (median exon inclusion 58% *vs* 24%), whereas 2 CUYG constructs were found among splicing spoilers. Secondary structure predictions of each mutated transcript suggested that as many as 252/256 (98.4%) tetraloops would maintain the canonical structure of the MIR hairpin, including the cytosine bulge at exon position 18 and the internal AAA triloop. Stable alternative structures, which were predicted for 11 of the tested 76 *FGB* pre-mRNAs, lacked the cytosine bulge (**Fig. 5B**); interestingly, these constructs were significantly associated with skipping of the MIR exon (32.9% *vs* 9.5%, *P* < 0.05; Mann-Whitney rank sum test). Similar to triloops, correlation between exon inclusion levels of tetraloop mutants and corresponding tetranucleotide frequencies in enhancers/silencers was highly significant (**Table S1**, *cf.* **Figs. 2D** **and S7A–C**).

### Splicing activities of terminal tetraloops and stem identity

To determine the extent to which the tetraloop sequence can predict exon inclusion levels in the presence of a distinct stem, we created a library of random tetraloops that cap the TSL1 hairpin in *SMN2* exon 7 (**Fig. S4A**). In the native context (TSL1/N; **Fig. S7D**), the TSL1 hairpin is formed by 6 Watson-Crick base-pairs terminating by the AGAC tetraloop.^21^ The 5′ loop-closing base is uracil at exon position 6 of *SMN2;* in contrast, cytosine at this position in the splice-proficient *SMN1* counterpart eliminates the loop-closing base-pairing, increases the loop size and shortens the stem to 5 base-pairs,^21^ the size of the upper stem of the MIR hairpin (**Fig. 1A**). Exon inclusion levels measured for 61 different TSL1/N tetraloops in *SMN2* reporters showed significant correlation with frequencies of matching tetranucleotides in splicing enhancers (**Fig. S7E**) and silencers (**Fig. S7F**). When the native loop-closing base pair U-A in TSL1 was replaced by G-C (TSL1/H, **Fig. 7G–I**), exon inclusion levels increased but the correlation was preserved even in the less informative context of the MIR-onized hairpin. Thus, as for triloops, tetraloop-closing base pairs accounted for a significant fraction of stem-mediated splicing activities.

**Figure 6.**
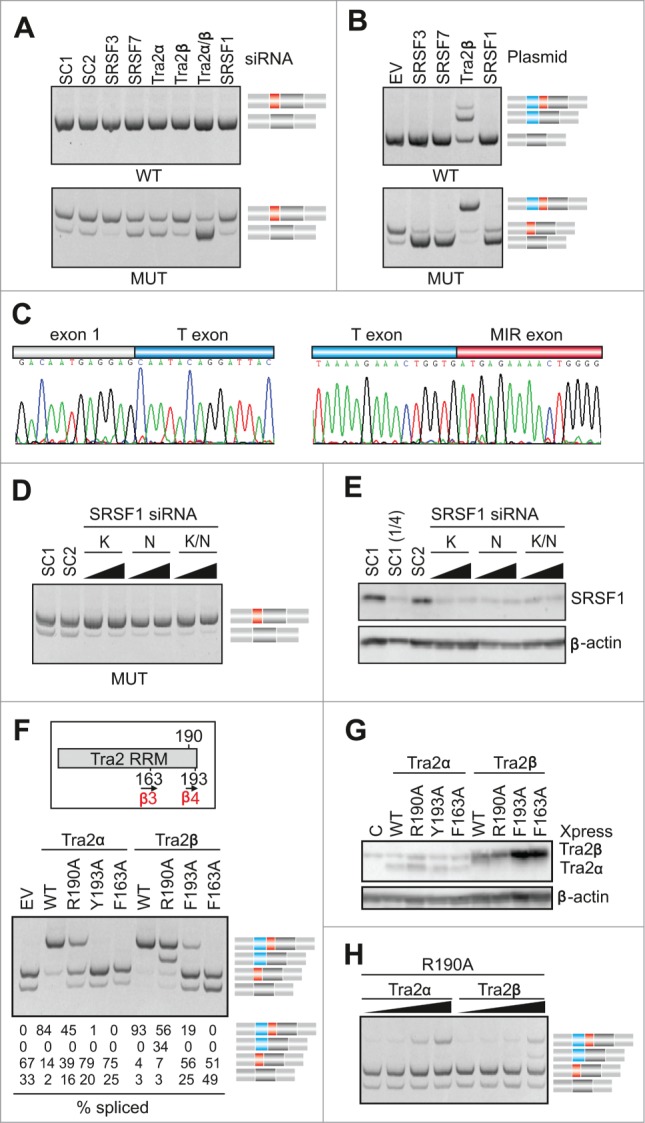
Tra2-induced activation of cryptic exons in *FGB* intron 1. (**A**) Activation of the MIR exon is promoted by Tra2. siRNAs targeting the indicated SR or SR-like proteins are at the top, spliced products to the right, *FGB* reporters are at the bottom. SC1, SC2, scrambled controls. MIR and T exons are denoted throughout as red and blue boxes, respectively. Their position in the pre-mRNA is shown in **Figure 1D**. SRSF1 and Tra2 immunoblots are shown in panels E and G; depletion levels of endogenous proteins were as described.^74,75^ (**B**) T exon activation in cells overexpressing Tra2β. Expression plasmids are at the top; EV, empty vector DNA. (**C**) Nucleotide sequence of transcripts with T and MIR exons. Sequenced RT-PCR products are color coded as in panel B. (**D**) Downregulation of SRSF1 promotes MIR exon inclusion. Final concentration and sequence of SRSF1-targeting siRNAs (termed K and N) was as described.^75,82^ (**E**) Western blot analysis with anti-SRSF1 antibodies. (1/4), a quarter of the lysate loaded to facilitate estimates of depleted proteins. (**F**) Distinct role of Tra2α and Tra2β residues R190 and Y/F193 in activation of adjacent exons. The *upper panel* shows relative positions of mutated residues in the indicated β-sheets of Tra2 RRMs, based on previously published alignments.^52,53^ The relative abundance of cryptic exons in cells overexpressing wild-type and mutated Tra2 proteins is in the *lower panel*. Tra2 substitutions are shown at the top. Spliced products (shown to the right) are quantified at the bottom. Concentration of reporter and expression plasmid DNA was 250 and 500 ng/mL, respectively. (**G**) Immunoblotting of HEK293 cell lysates using antibodies against β-actin and Xpress. Tra2 mutations are at the top. C, no plasmid DNA control. (**H**) Dose-dependent activation of T- and MIR exons in cells expressing Tra2α R190A and Tra2β R190A. Final DNA concentrations of plasmid expressing Tra2α and Tra2β were 30, 100, 300 and 600 ng/mL, and 6, 20, 60 and 180 ng/mL, respectively.

**Figure 7.**
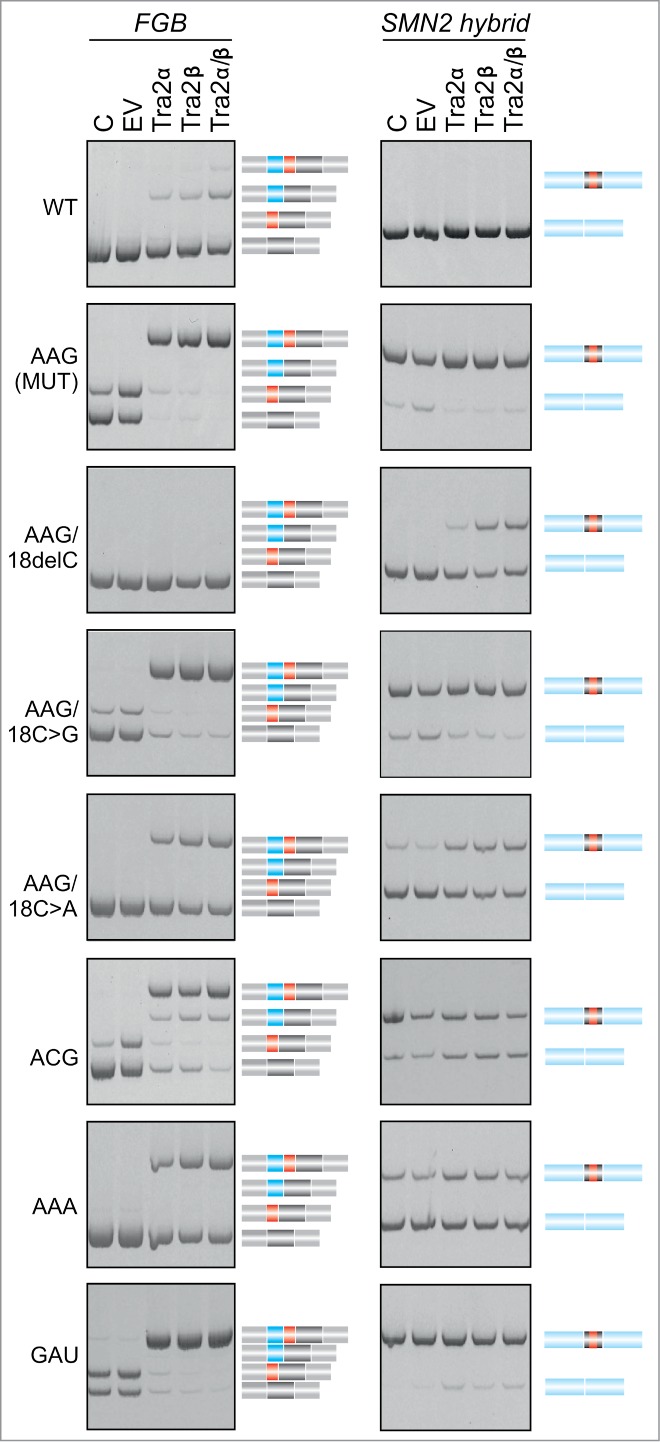
Tra2-induced cryptic exon activation is controlled by the MIR hairpin located >1.5 kb downstream. *FGB* and hybrid *SMN2-MIR* constructs were cotransfected with Tra2 expression plasmids and controls (top). The *FGB* pre-mRNA is shown in **Figure 1D** and the hybrid *SMN2-FGB* pre-mRNA in **Figure 4A**. Spliced products are shown schematically to the right; exons are color -coded as in the pre-mRNAs. Mutated splicing reporters are to the left; mutations were introduced in the terminal triloop (exon position 24–26)/bulge (exon position 18) of the MUT construct, as indicated. WT, wild construct without the 23A > G substitution; EV, empty vector; C, reporter-only controls.

### Tra2-induced activation of cryptic exons in *FGB* intron 1

To identify ligands that control MIR exon inclusion and their interactions with the hairpin, we first examined MIR exon splicing in cells depleted of known splicing factors, including serine/arginine-rich (SR) proteins as important regulators of exon inclusion.^51^ Screening with siRNAs targeting >20 factors (data not shown) revealed significant exon skipping in cells depleted of the human homolog of *Drosophila transporter* (Tra2) and in cells transfected with siRNAs targeting SR protein SRSF7 (also known as 9G8; **Fig. 6A**). Interestingly, overexpression of Tra2, but no other tested splicing factors or controls, activated an upstream exon (termed T, for Tra2-activated; **Figs. 1A, 6B and C**). The T exon contained optimal Tra2β binding sites ^52,53^ and was about twice as long as the MIR exon (**Fig. S8**). In contrast to Tra2, down-regulation of SR proteins SRSF3 (SRp20) and SRSF1 (SF2/ASF) promoted exon inclusion while their overexpression increased exon skipping (**Figs. 6A–E**). For a subset of SR proteins, we also found significant correlation between MIR exon inclusion and scores for their putative exonic binding sites, including SRSF1 and SRSF2 (**Fig. S9**).

The RNA recognition motif (RRM) of Tra2β binds GAA- and CAA-containing sequences through hydrogen-bond formation with several amino acids as well as through stacking interactions mediated by unusually aligned rings on the β-sheet surface.^52,53^ In a solution structure of RRM-Tra2β/(GAA)_2_, the protein contacted the A_3_G_4_A_5_A_6_ tetramer through arginine 190 (A_3_G_4_) and phenylalanines 193 (A_5_) and 163 (A_5_A_6_).^52^ Cotransfection of our *FGB* MUT reporter with expression plasmids encoding Tra2α or Tra2β that had R190A, Y/F193A and F163A substitutions revealed that exon T activation was completely abrogated by mutation F163A (**Fig. 6F**), indicating that this β3 residue is critical for T exon inclusion in each protein. However, mutations of β4 amino acids in the 2 proteins produced a distinct splicing pattern. Most notably, Tra2β R190A was capable of activating transcripts that contained the T exon but lacked the MIR exon, whereas Tra2α R190A promoted only transcripts with both exons. This was further supported by addition of increasing amounts of each R190A plasmid to cell cultures to exclude that the distinct activation of adjacent exons is due to a higher expression of Tra2β over Tra2α and by immunoblots confirming expression of each mutated construct (**Figs. 6G, H**). We conclude that MIR and T exons have distinct requirements for the C-terminal extension of Tra2 RRMs.

To test if the triloop identity in the MIR hairpin can influence T exon activation, we transfected WT and MUT versions of *FGB* and hybrid *SMN2-FGB* reporter plasmids into cells overexpressing Tra2α and/or Tra2β and compared inclusion levels of their mid-exons (**Fig. 7**). Interestingly, Tra2-induced T-exon activation was observed in the WT FGB clone but ‘T-exon only’ transcripts disappeared in the context of the AAG and other tested triloops, except for ACG. Even more remarkably, the Tra2-induced activation of the T exon was completely abrogated in *FGB* by deletion of the bulged cytosine at position 18 of the MIR exon and was also reduced by substitutions of this residue. The same changes also diminished activation of *SMN2-FGB* exon 7 (**Fig. 7**).

Collectively, these data identify proteins that control inclusion of the MIR exon in mature transcripts and reveal a Tra2-activated cryptic exon >1.5 kbp upstream whose selection was dramatically affected by single-stranded regions of the MIR hairpin. Finally, they show a distinct role of C-terminal residues in Tra2α and Tra2β RRMs in activation of the 2 adjacent exons.

### Tra2β, SRSF1 and hnRNPs A1 and H bind to hybrid *FGB* RNAs

**Figure 8.**
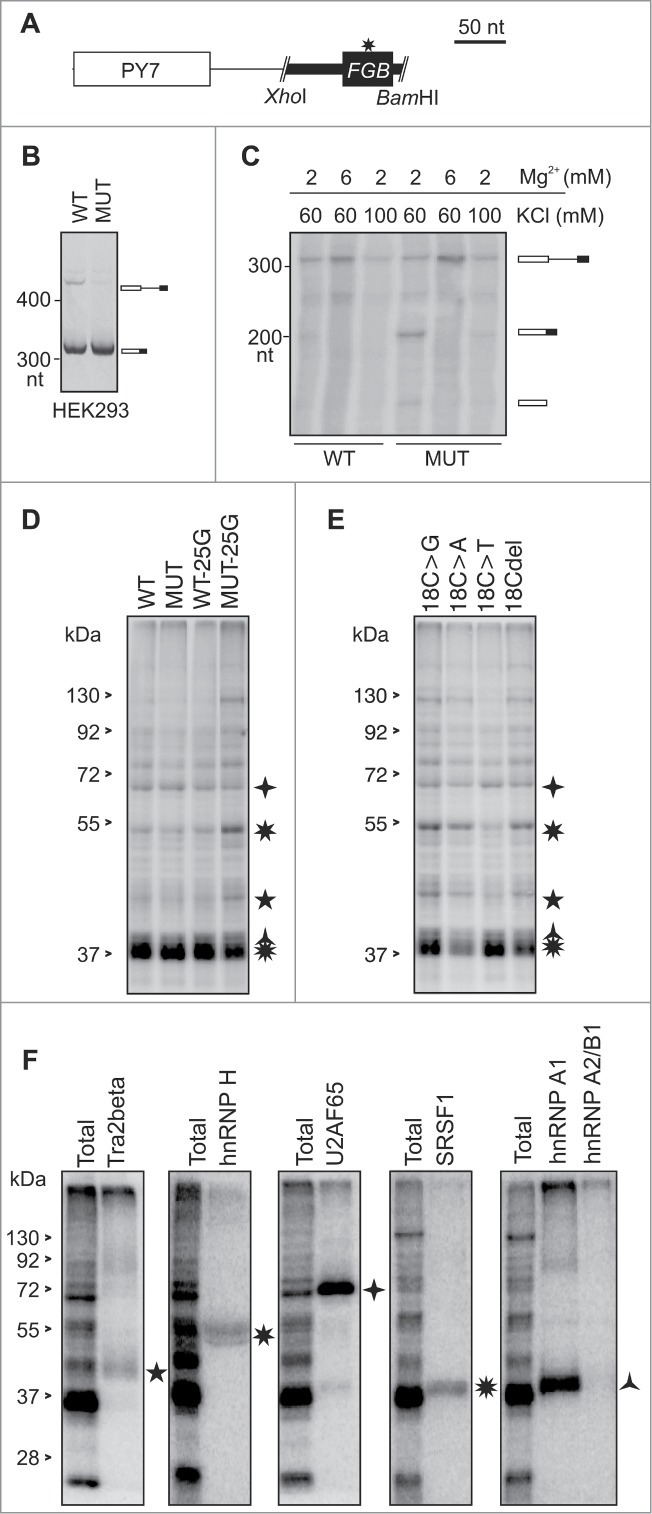
**Identification of proteins that bind to *PY7-FGB* transcripts** (**A**) Schematics of hybrid *PY7-FGB* reporter construct for *in vitro* splicing. *PY7* ^54^ and MIR (**Figure 1D**) exons are represented by white and black rectangles (to scale at the top), respectively. The A > G mutation is shown by an asterisk. Cloning primers are in **Table S3**. (**B**) Splicing of pCR-*PY7-FGB* hybrid constructs in HEK293 cells. Spliced products are shown to the right. (**C**) *In vitro* splicing of WT and MUT *PY7-FGB* hybrids in HeLa nuclear extracts. ^32^P-UTP labeled RNAs were incubated in HeLa nuclear extracts for 1 hr. Concentrations of Mg^2+^ and KCl are at the top, spliced products are to the right. The size of the pre-mRNA/mRNA was 327/201 bp. **(D, E)** UV-crosslinking of proteins to ^32^P-UTP-labeled WT and MUT transcripts and their mutated versions. Asterisks correpond to proteins identified by immunoprecipitation in panel F. Panel E shows differential crosslinking to ^32^P-CTP-labeled bulge mutants at MIR exon position 18. (**F**) Immunoprecipitation of the crosslinked proteins with the indicated antibodies.

To identify proteins that bind to the MIR hairpin, we subcloned a 99-nt amplicon containing the MIR exon and flanking sequences into a *PY7* reporter that is efficiently spliced in nuclear extracts^54^ (**Fig. 8A**). Similar to the canonical *FGB* minigene (**Fig. 1D**), the MUT version of the hybrid *PY7-FGB* reporter subcloned into pCR3.1 was spliced more efficiently *in cellulo* than the WT counterpart (**Fig. 8B**). *In vitro* splicing of WT and MUT *PY7-FGB* transcripts showed that both alleles were spliced at optimized conditions at 2 mM Mg^2+^/60 mM KCl, but the MUT *PY7-FGB* construct was spliced more efficiently than the WT *PY7-FGB* hybrid (**Fig. 8C**). Additional 11 mutated *FGB* constructs that were subcloned into *PY7* revealed positive correlation between the percentage of splicing in nuclear extracts and MIR exon inclusion levels upon transfection into HEK293 cells (*r* = 0.55, *P* < 0.05, t-test, data not shown), indicating that the MIR hairpin contributes significantly to splicing efficiency of the hybrid pre-mRNA substrate.

Disappointingly, UV-RNA crosslinking of *PY7-FGB* reporters under splicing conditions did not reveal a distinct pattern of bound proteins between WT and MUT transcripts with either ^32^P-UTP or ^32^P-CTP (lane 1 and 2, **Fig. 8D**, data not shown). As compared to controls, however, we observed lower signal intensity from ∼38–40 kD bands and stronger signal from ∼45 and ∼55 k_D_ proteins for transcripts that contained an A > G substitution at exon position 25 of the MUT construct (lane 4, **Fig. 8D**, data not shown). This AAG > AGG triloop substitution diminished inclusion of the MIR exon (**Fig. 2A**, **Table S4**). Proteins of the same size range also differentially bound transcripts derived from cytosine bulge mutants (**Fig. 8E**). Immunoprecipitation of crosslinked proteins revealed that apart from a control U2AF65/35, the transcript was bound by Tra2β, SRSF1 and heterogenenous ribonucleoproteins (hnRNPs) H and A1 (**Fig. 8E, F**).

## Discussion

Using a novel, single-hairpin exon selection model, we have shown that short and exposed RNA loops are key components of the structural splicing code and account for most exon-repressing/-promoting activities of splicing silencers/enhancers, demonstrating a pervasive role of the most common RNA structural motifs in exon recognition. Our results strongly indicate that these structures act as principal exon selection modules (**Fig. 4A–E**, **Fig. S7**) that control activation of adjacent exons (**Fig. 7**), with exposed loops and bulges providing key exon recognition contacts. They also show that intramolecular base-pairing contributes significantly to the mysterious context dependency of auxiliary splicing signals (**Fig. 4F, G**).

The observed correlation between free energies of MIR hairpin mutants and their exon inclusion levels (**Fig. 1E, 4G**) is consistent with a higher exon inclusion by stem-loop destabilization previously noticed for *MAPT*,^55^ *SMN2*,^17^ *FN1*,*^13^* *PSEN2* ^56^ and *TNNT2* ^57^ and also with decreased stability of secondary structures of exonized *Alu*s as compared to their non-exonized counterparts.^42^ The size of the MIR hairpin corresponds to the most common range of human RNA folds predicted using genome-wide approaches, with many thousands estimated to exist in introns.^58^ The MIR upper stem shows similarities to structures previously found to promote exon inclusion, including a 4-nt upper stem interrupted by a single uridine bulge and terminating in a tetraloop in *PS2* exon 5 ^56^ or a 5-nt upper stem with a single adenine bulge, terminating in a well-characterized purine-rich ESE in *FN1* exon ‘EDA’.^13^ The MIR hairpin is also similar to the IRE, a regulatory conserved stem-loop increasingly found in mRNAs encoding important proteins with diverse funct-ions ^44,45,59^ (**Fig. S10**). IREs also have a 5-nt upper stem and a flexible cytosine-type bulge, which was shown to bind domain 4 of iron regulatory protein 1.^60,61^

Secondary structure predictions are generally more accurate for inverted repeats than average pre-mRNAs. Inverted repeats are significantly enriched in eukaryotic genomes, suggesting that they are functionally important.^43,62,63^ The majority of human inverted repeats are derived from SINEs.^43^ These elements should therefore provide the most attractive structural models of mammalian pre-mRNA splicing, alleviating uncertainties in accuracy of RNA secondary structure predictions encountered with typical pre-mRNAs. This notion is consistent with a low fraction of predicted alternative foldings with a similar energy, both for triloop (8/64, 12.5%) and tetraloop (11/76, 14.5%) *FGB* mutants. Such models should also facilitate identification of RNA-protein interactions across their exonized sequences. Proteins that influenced MIR exon inclusion (**Figs. 6**–**8**) may be engaged in interactions across the hairpin, perhaps similar to a cooperative assembly of Tra2/SRSF7 on a purine-rich enhancer in *Drosophila doublesex*.^64^ **Fig. 6** also suggests that Tra2 is a stronger splicing activator than other SR protein family members, confirming earlier observations ^65^; in addition, optimal binding sites for Tra2β ^52,53^ were enriched among high-inclusion triloops (**Fig. 2A**). Future studies should therefore explore the role of proteins that recruit Tra2 to MIR RNA, including hnRNP G, which was recently shown to contact diadenosines in single-stranded regions.^66^

As shown for *SMN2* exon 7,^67^ SSOs that bind exonic sequences and promote their inclusion in mRNA are relatively uncommon. For example, exon-promoting antisense targets were found only for 2/10 (20%) tested oligos in the initial walk across *SMN2* exon 7.^67^ By analogy, we found only 2 intron retention-repressing SSOs among 20 (10%) initially tested across *INS* intron 1.^68^ Our study revealed that exon-promoting and -repressing effect of the SSO targeting a more accessible side of the MIR hairpin is controlled by the flanking triloop sequence (**Fig. 3**). This observation has important implications for potential clinical use of antisense reagents and for personalized medicine because even optimized SSOs may produce unintended outcomes if the target is surrounded natural variants in the vicinity that have not been tested. Also, distance between such natural variants and the antisense target might be much longer than that observed for the MIR hairpin, highlighting the requirement for structural design of SSOs.

Finally, clone resources developed in this work will be helpful for systematic studies that investigate ligand preferences and their requirements for splicing outcomes. Reference exon inclusion levels for tri- and tetra-loop sequences capping a stable stem (**Figs. 2A, 5A**, **S2A and Table S4**) will be useful for studying auxiliary splicing motifs in other hairpins and their long-range interactions. Identification of distant contacts between the MIR element and upstream exon (**Fig. 7**)^27,69,70^ will facilitate our understanding of tertiary interactions in splicing regulation. Splicing-proficient triloops were rich in diadenosines (**Fig. 2A**) and could thus be engaged in more extensive RNA tertiary interactions than splicing spoilers. Diadenosines are frequently involved in formation of the most common 3 dimensional motifs.^71^ This hypothesis is also supported by the observed promotion of the MIR exon inclusion by the 5′ stem SSO, which required AA-containing triloops (**Fig. 3**). As shown for group II self-splicing introns,^72^ adoption of complex tertiary structures could remain very important also for splicing catalysis of their likely evolutionary descendants, i.e., human introns. Our model should therefore prove useful for evaluating the relative importance of diadenosine binding proteins ^66^ and intramolecular tertiary contacts in RNA processing. Ultimately, high resolution structures will help us understand how hairpins facilitate exon selection and alternative splicing, which should substantially improve future iterations of exonic and intronic splicing code, accurate prediction of phenotypic consequences of disease-causing splicing mutations and their correction by antisense strategies.

## Materials and Methods

### Splicing reporter constructs

To facilitate mutagenesis, the *Bam*HI-*Xho*I fragment of pcDNA-FGB-V5-His-TOPO (a gift of Dr Ryan Davis, University of Otago)^46^ was subcloned into pCR3.1 (Invitrogen). The *Hin*dIII site was removed from the pCR3.1 polylinker using a *Nhe*I/*Bam*HI digest and Klenow enzyme treatment. Mutagenesis of the resulting minigene (termed pCR3.1-*FGB*) was carried with 673-bp products amplified by cloning primers C-F and C-R (**Fig. 1D**, **Table S3**). Mutagenic PCR primers for tri- and tetra-loops were degenerated at each loop position. PCR products were digested by *Kpn*I/*Hin*dIII, subcloned into pCR3.1-*FGB* and propagated in a *E. coli* strain DH5α. Each plasmid DNA was completely sequenced using primer S (**Table S3**) to confirm desired mutations and exclude PCR-introduced errors. Reporter constructs with and without the disease-causing A > G substitution are referred as MUT and WT, respectively, throughout the text.

Hybrid reporter constructs *SMN2-FGB, F9-FGB* and *L1CAM-FGB* were prepared by introducing a 24-bp *FGB* hairpin into central exons of previously published minigenes.^17,73^ The *SMN2* minigene^17^ was a gift from Drs Ravi and Nathalia Singh, Iowa University. The *F9* minigene had a substitution of the branch point adenine^73^ to obtain similar levels of exon inclusion and skipping. The *L1CAM* minigene^73^ contained the MIR hairpin at 2 different positions of the central exon to create reporters *L1CAM(r1)-FGB* and *L1CAM(r2)-FGB.* Cloning primers for hybrid reporters were *SMN2*-*FGB*-F and *SMN2-FGB*-R, *F9-FGB*-F and *F9-FGB-*R, *L1CAM(r1)-FGB-*F1 and *L1CAM(r1)-FGB*-R1, and *L1CAM(r2)-FGB-F2* and *L1CAM(r2)-FGB*-*R2,* while triloop mutants were obtained using primers *SMN2*-NNN, *F9*-NNN, *L1CAM*(*r1*)-NNN and *L1CAM(r2)-*NNN (**Table S3**).

To prepare constructs for *in vitro* splicing and UV crosslinking (*PY7-FGB*), we amplified a 99-bp segment encompassing the cryptic exon and flanking MIR sequences using primers *PY7*-*FGB-*F-*Xho*I and *PY7*-*FGB*-R-*Bam*HI (**Table S3**). PCR products were digested with *Xho*I/*Bam*HI and subcloned into an *in vitro* splicing-proficient clone PY7^54^ (a gift from Professor Christopher Smith, University of Cambridge). Minigene pCR-*PY7-FGB* (pCR3.1, LifeTechnologies) for *in vivo* splicing was prepared with primers pCR-PY7F-*Nhe*I and *PY7*-*FGB*-R-*Bam*HI (**Table S3**) using the *PY7-FGB* plasmid DNA as a template. Spliced products were amplified using primers PL1 and PL2 (**Table S3**).

Mammalian expression plasmids encoding Tra2 proteins were obtained by PCR employing the *Pfu* polymerase, primers shown in **Table S3** and cDNA as a template. PCR products were first cloned between *Bam*HI and *Not*I sites of pcDNA3.1-HisC (Invitrogen). To increase the intron-mediated expression of Tra2 proteins and move the Xpress tag to the N-terminal, plasmids were digested using *Nhe*I and *Not*I and inserted into the pCI-neo vector (Promega). Mutated Tra2 versions were prepared by overlap-extension PCR using wild-type pCI-neo Tra2 DNA and mutagenic primers Tra2α-F163A, Tra2β-F163A, Tra2α-Y193A, Tra2β-F193A, Tra2α-R190A and Tra2β-R190A (**Table S3**). All clones were sequenced to confirm the identity of intended mutations and exclude undesired changes.

### Cell cultures and transfections

HEK293, HeLa, HepG2 and COS7 cell lines were cultured under standard conditions as described previously.^74^ Transient transfections were performed in 12- or 24-well plates using FuGENE (Roche) according to manufacturer's recommendations. Final concentration of each plasmid DNA in the culture medium was 500 ng/mL. Cells were harvested 24 hours post-transfection for RNA extraction, as described.^74^

### Detection and measurements of spliced products

Total RNA was isolated using TRI-reagent (Ambion) according to manufacturer's recommendations and reverse-transcribed using the Moloney murine leukemia virus reverse transcriptase (Invitrogen) and the oligo-d(T) primer, essentially as described.^74^ For RT-PCR, we used vector primers PL1 and PL2 (**Table S3**). To amplify spliced products of *SMN2,* we used primers P1 and P2, as reported previously.^47^ Amplifications were for 28 cycles. PCR products were separated on polyacrylamide gels and exon inclusion levels were measured as described.^39,74^

### Splice-switching oligonucleotides (SSOs) and small interfering RNAs (siRNAs)

The WT and MUT reporters were individually cotransfected with SSOs targeting the loop, 5'stem and 3'stem in the MIR hairpin. SSOs (MWG Biotech) were 2′-*O*-methyl-modified at each sugar residue and uniformly labeled with phosphorothioates. Oligoribonucleotides targeting loops (SSO-AAG and SSO-GAU) and the 5′ and 3′ stem are in **Table S3**. Spliced products were detected and measured as described above. Sequences of siRNAs targeting splicing factors were described previously.^75^

### Western blot analysis

Cells (mock)-transfected with siRNA and expression plasmids were washed twice with PBS and immediately lysed in the RIPA buffer (New England Biolabs).^75^ Protein lysates were loaded on to 10% SDS-PAGE and transferred to polyvinyl difluoride membranes (Amersham) using electroblotting. Membranes were incubated with the following antibodies: Xpress (Invitrogen, R910-25), SFRS7 (9G8; Sigma, SAB1101226), Tra2β (Abcam, ab31353), SRSF1 (Abcam, ab38017), actin (Abcam, ab37063) and horseradish peroxidase-conjugated secondary antibodies. Proteins were detected using the Immun-Star WesternC kit (Biorad).

### *In vitro* splicing assay

The *PY7-FGB* substrates were transcribed using the MAXIscript kit (Ambion) with *Bam*HI-linearized plasmids as templates. *In vitro* transcription reaction contained 1x transcription buffer, 1 μg of linear DNA, 60 μCi of [α-^32^P]UTP or [α-^32^P]CTP (800 Ci/mmol, PerkinElmer), 0.5 mM ATP, 0.5 mM CTP or 0.5 mM of UTP, 0.1 mM GTP, 0.4 mM m7G(5′)ppp(5′)G, 1 U/μL of ribonuclease inhibitor (Promega), and 2U/μL of SP6 RNA polymerase. Transcription reactions were incubated at 37°C for 1 and 3 hrs. Internally labeled transcripts were gel purified using PAGE.

Each splicing reaction contained 0.1 nM of RNA (∼10ng), 30% of HeLa nuclear extract (4C Biotech), 1 mM DTT, 60 mM KCl, 12 mM Hepes–KOH, pH 7.9, 0.5 mM ATP, 20 mM creatine phosphate, 2 mM MgCl_2_, 0.25 U/μL of ribonuclease inhibitor, and 2.6 % of polyvinylalcohol. Following incubation at 30°C for 2 hours, 100-μL splicing reactions were digested with proteinase K (100 mM Tris, pH 7.5, 1% SDS, 150 mM NaCl, 10 mM EDTA, 0.05 μg/μl tRNA, and 0.25 mg/mL proteinase K) at 37°C for 15 min, extracted with phenol and chloroform, precipitated with ethanol and separated using PAGE. Gels were dried and exposed to a Typhoon PhosphorImager (Molecular Dynamics).

### UV-RNA crosslinking and immunoprecipitation

Binding reactions were the same as *in vitro* splicing, except for omission of polyvinylalcohol. Reactions were irradiated with 254-nm UV light (Ultralum) on ice for 10 min and treated with a mixture of RNase A (0.05 U/1 μl) and RNase T1 (2U/μl; Ambion) at 30°C for 20 min. An equal volume of 2x Laemmli buffer was added and samples were boiled for 5 min before loading on to 10% SDS-PAGEs. The gels were fixed, dried and exposed to PhosphorImager screens. For immunoprecipitation, 50 μl of cross-linked reactions were mixed with 400 μl of the IP buffer (50 mM Tris-HCl, pH 8.0, 150 mM NaCl, 5 mM EDTA, 0.05% NP-40), 50 μl of protein G-coupled Sepharose beads and the indicated antibodies. Antibodies were purchased from Sigma (U2AF65, hnRNP A2/B1), ABcam (Tra2β, SRSF1) or were a generous gift from Professors Douglas Black (hnRNP H), UCLA, and Gideon Dreyfuss (hnRNP A1), University of Pennsylvania. After incubation at 4°C for 2 hrs, the samples were washed 5 times with the IP buffer. Beads were resuspended in 20 ul of the Laemmli buffer, boiled for 5 min and loaded on a 10% SDS-PAGE.

### Tri- and tetra-nucleotide frequencies in splicing silencers and enhancers

These data were computed for previously defined RESCUE exonic splicing enhancers (ESEs),^4^ FAS exonic splicing silencers (FAS-ESSs),^5^ putative exonic splicing enhancers and silencers (PESSs and PESEs),^76^ exon identity elements (EIEs),^7^ QUEPASA ESEs/ESSs,^9^ neighborhood inference (NI) ESEs/ESSs^77^ and exon regulatory sequences (ERSs)^6^ (**Table S1**). Frequencies of tri- and tetra-nucleotides in these motifs were computed using Microsoft Excel functions or VBA scripts (available on request).

### Circular dichroism (CD) and nuclear magnetic resonance spectroscopy (NMR)

RNA oligo for CD and NMR were purchased from Thermo Scientific, deprotected according to manufacturer's instructions, lyophilised and stored at −20°C. Stock solutions were prepared from the desalted, lyophilised samples by resuspending in milliQ water or KCl buffer (100 mM KCl, 10mM K_2_HPO_4_/KH_2_PO_4_, pH 7.0, milliQ water) to a final concentration of 2–4 μM.

CD spectra were acquired using a PiStar-180 spectrophotometer (Applied Photophysics Ltd, Surrey, UK), equipped with a LTD6G circulating water bath (Grant Instruments, UK) and thermoelectric temperature controller (Melcor, USA). Samples were heated in the cell to 95°C for 15 minutes and samples were then annealed by cooling to room temperature over a period of 4 h. CD spectra were recorded over a wavelength range of 215–340 nm using a 1 cm path length strain-free quartz cuvette and at the temperatures indicated. Data points were recorded at 1 nm intervals. A bandwidth of 3 nm was used and 5,000 counts acquired at each point with adaptive sampling enabled. Each trace is shown as the mean of 3 scans (±SD ). CD temperature ramps were acquired at 265 nm corresponding to the band maxima of the folded hairpin structure. Ranges between 5 and 95°C were used, with points acquired at 0.5°C intervals with a 120–180 second time-step between 0.5°C increments. Points were acquired with 10,000 counts and adaptive sampling enabled. Heating and cooling studies were compared to check for hysteresis and overall reversibility.

NMR spectra (^1^H) were collected at 800 MHz using a Bruker Avance III spectrometer with a triple resonance cryoprobe. Standard Bruker acquisition parameters were used. Data were collected using Topspin (v. 3.0) and processed in CCPN Analysis (v. 2.1).

### Triloop database

Triloop frequencies of previously determined RNA stem-loop structures were determined for a total of 823 triloops in 123 small subunit rRNAs, 223 large subunit rRNAs, 309 5S rRNAs, 484 tRNAs, 91 signal recognition particles, 16 RNase P RNAs, 100 group I introns and 3 group II introns ^48^ (and Brent Znosko, personal communication). The triloop database was considered to be representative of triloops found in naturally occurring RNAs.^48^

### RNA secondary structure predictions

Overlapping segments of *FGB* intron 1 and all mutated transcripts were used as an input into RNAstructure (v. 5.2, default options),^78^ which accommodates a dynamic programming algorithm on the principle of minimizing free energy.^79^ We used the same input also for Mfold ^80^ and RNAfold from the Vienna RNA suite ^81^ to confirm secondary structure predictions.

### Statistical analysis

Pearson correlation coefficients and Mann-Whitney rank sum tests were computed using SigmaStat (v. 3.5; Systat Software). Kruskall-Wallis and Friedman tests were computed using SigmaStat or Stat200 (Biosoft, UK). Correlation coefficients were compared using Fisher *r*-to-*z* transformations. Weighted contribution of each base (*N*) to cryptic splicing (*f,* ***Fig. 2A***) for *n* triloops was calculated as

$$f_{N}=100\sum_{1}^{n}\left(k_{n}N_{n}\right)/\sum_{1}^{n}\left(k_{n}\right)$$

where *k* is the mean exon inclusion level of 2 independent transfections.
