# Supplementary material for: The role of short RNA loops in recognition of a single-hairpin exon derived from a mammalian-wide interspersed repeat
Source: RNA Biol. 2015 Mar 31;12(1):54–69. doi: 10.1080/15476286.2015.1017207 (PMC4615370; doi:10.1080/15476286.2015.1017207)
Supplement: Supplemental Materials [file krnb-12-01-1017207-s001.pdf]

SUPPLEMENTARY FIGURES AND TABLES

The role of short RNA loops in selection of a single-hairpin exon derived from a mammalian-wide interspersed repeat

Jana Kralovicova[1], Alpa Patel[2], Mark Searle[2,] Igor Vorechovsky[1]

[1]University of Southampton Faculty of Medicine, Southampton, United Kingdom

[2]University of Nottingham, Centre for Biomolecular Sciences, School of Chemistry, Nottingham, NG7 2RD, United Kingdom

SUPPLEMENTARY FIGURES

A

```
FGB intron 1     1382 TATAGTCAACTGGTTAAA--CAGGAAAATCTGGAACCAGCCTGGCTGGGT 1429
                           ivv v       i--  v i v-      i    v   v
MIR consensus       5 TATAGCATAGTGGTTAAGAGCACGGAC-TCTGGAGCCAGACTGCCTGGGT 53

FGB intron 1     1430 TTTAATCTTAGC---ACCATCCTACTAAATGT 1458
                       iv    iii  ---i   - i     iv
MIR consensus      54 TCGAATCCCGGCTCTGCCA-CTTACTAGCTGT 84
```

B

```
FGB intron 1     1937 TAATGAGCACTTATTAT-TGCCAAGTACTGTTCTGAGGGTACCATATGCA 1985
                        v     i    i   -     i i        i  v ivii i   i
MIR consensus     246 TATTGAGCGCTTACTATGTGCCAGGCACTGTTCTAAGCGCTTTACATGTA 197

FGB intron 1     1986 ATAAGTTATTTAATCCTTACAATAATCTTGTAAGGCAGATTCAAACTATC 2035
                      v   v i          i    i  i i i i   i  i----     i
MIR consensus     196 TTAACTCATTTAATCCTCACAACAACCCTATGAGGTAGGT----ACTATT 151

FGB intron 1     2036 ATTACACTTATTTTACAGATGAGAAAACTGGGGCACAGATA----AAGCA 2081
                          iv ii              i      i        v ----   i
MIR consensus     150 ATTATCCCCATTTTACAGATGAGGAAACTGAGGCACAGAGAGGTTAAGTA 101

FGB intron 1     2082 ACTTGCCCAAGGTCTCATAGCT-GTAAGT--CAACCCTACGGTCAAGACC 2128
                                    v  i    -      --  ivv iiv i ivii
MIR consensus     100 ACTTGCCCAAGGTCACACAGCTAGTAAGTGGCAGAGCCGGGATTCGAACC 51

FGB intron 1     2129 TACAAGTAGCCGAGCTCCAGAGTACAT 2155
                      --  i i  i vi          v i
MIR consensus      50 --CAGGCAGTCTGGCTCCAGAGTCCGT 26
```

Figure S1

Nucleotide sequence alignment of *FGB* intron 1 MIR elements and the MIR consensus

(A) Sense MIR element. **(B)** Antisense MIR element. Alignments were created using the sensitive mode of the RepeatMasker Web server (http://www.repeatmasker.org/cgi-bin/WEBRepeatMasker), version 3.2.9.; i, transitions; v, transversions. The MIR exon is highlighted in grey, putative branch points in yellow. Branch points were predicted using a support vector machine algorithm [1]. The 24-bp hairpin is underlined.

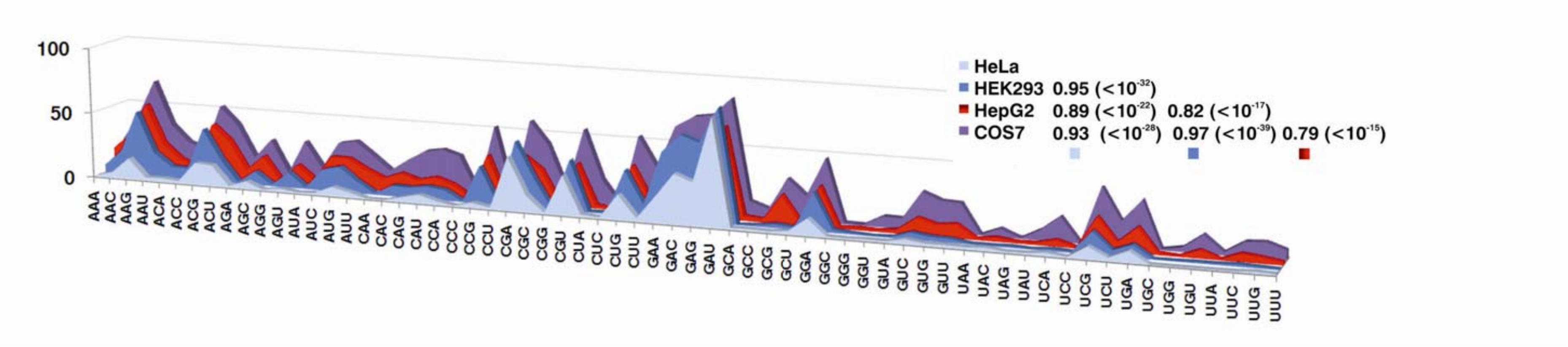

Figure S2
Exon inclusion landscape of a MIR hairpin with 64 terminal triloops in 4 cell lines

Triloop mutants are ordered alphabetically. Exon inclusion is in %. *P* values for the indicated Pearson correlation coefficients are shown in parentheses.

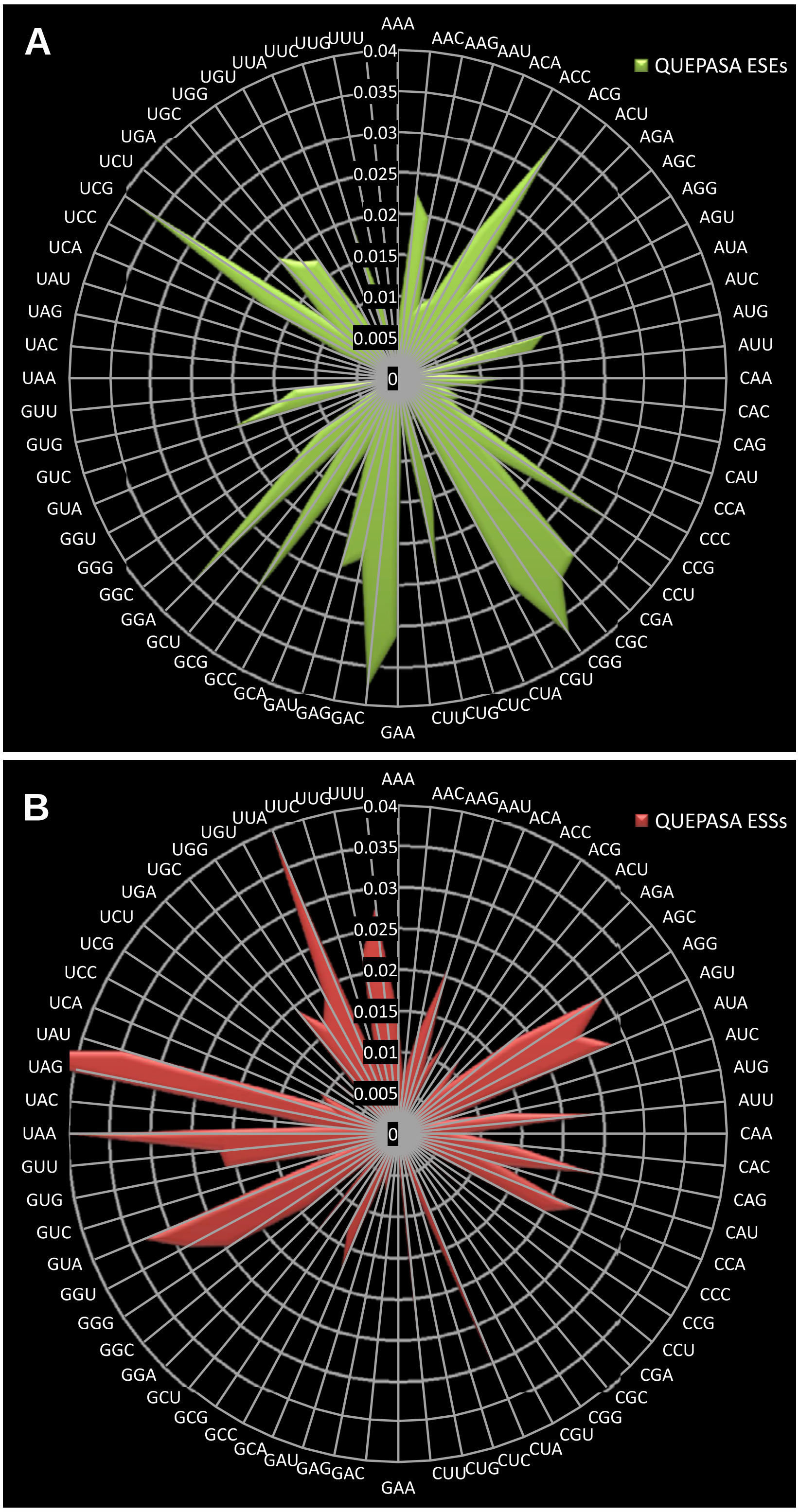

Figure S3

Trinucleotide frequencies in QUEPASA enhancers (A) and silencers (B)

MIR exon inclusion levels of corresponding triloop mutants are shown in Fig. 2A and Table S4.

A

Wild-type *SMN2* exon7
TSL1 TSL2
GGUUUUAGACAAAAUCAAAAAGAAGGAAGGUGCUCACAUUCCUUAAAUUAAGGA

***SMN2-FGB* WT**
GGUUUUAGACAAAAUCAAAAAGGGGCACAGAUAAAGCAACUUGCCCAAAUUAAGGA

***SMN2-FGB* MUT**
GGUUUUAGACAAAAUCAAAAAGGGGCACAGAUGAAGCAACUUGCCCAAAUUAAGGA

B

Wild-type *F9* exon 3
ACUGAAUUUUGGAAGCAGUAUGUUG

***F9-FGB* WT**
ACUGAAUUUUGGGGCACAGAUAAAGCAACUUGCCCAAGCAGUAUGUUG

***F9-FGB* MUT**
ACUGAAUUUUGGGGCACAGAUGAAGCAACUUGCCCAAGCAGUAUGUUG

C

Wild-type *L1CAM* exon 18 (replacement 1)
ACCCCCAGGCAAUCCCUGAGCUGGAAGGCAUUGAAAUCCUCAACUCAAGUGCCGUGCUGGUCAAGUGGCGGCCGGUGGACCUGGCCCAGGUCAAGGGCCACCUCCGCGGAUACAAU

***L1CAM-FGB* WT (replacement 1)**
ACCCCCAGGCAAUCCCUGAGCUGGGGCACAGAUAAAGCAACUUGCCCAAGUGCCGUGCUGGUCAAGUGGCGGCCGGUGGACCUGGCCCAGGUCAAGGGCCACCUCCGCGGAUACAAU

***L1CAM-FGB* MUT (replacement 1)**
ACCCCCAGGCAAUCCCUGAGCUGGGGCACAGAUGAAGCAACUUGCCCAAGUGCCGUGCUGGUCAAGUGGCGGCCGGUGGACCUGGCCCAGGUCAAGGGCCACCUCCGCGGAUACAAU

Wild-type *L1CAM* exon 18 (replacement 2)
ACCCCCAGGCAAUCCCUGAGCUGGAAGGCAUUGAAAUCCUCAACUCAAGUGCCGUGCUGGUCAAGUGGCGGCCGGUGGACCUGGCCCAGGUCAAGGGCCACCUCCGCGGAUACAAU

***L1CAM-FGB* WT (replacement 2)**
ACCCCCAGGCAAUCCCUGAGCUGGAAGGCAUUGAAAUCCUCAACUCAAGUGCCGUGCUGGUCAAGUGGCGGCCGGUGGGGGCACAGAUAAAGCAACUUGCCCUCCGCGGAUACAAU

***L1CAM-FGB* MUT (replacement 2)**
ACCCCCAGGCAAUCCCUGAGCUGGAAGGCAUUGAAAUCCUCAACUCAAGUGCCGUGCUGGUCAAGUGGCGGCCGGUGGGGGCACAGAUGAAGCAACUUGCCCUCCGCGGAUACAAU

Figure S4
Nucleotide sequences of hybrid exons

(A) *SMN2* exon 7. **(B)** *F9* exon 3. **(C)** *L1CAM* exon 18. Swapped segments are underlined. The A>G substitution leading to activation of the MIR exon in *FGB* is in red; terminal triloop mutated in each hybrid exon is highlighted in yellow. The number of sequence-verified triloop mutations was 64 for each hybrid. *SMN2* sequences forming TSL1 and TSL2 hairpins are highlighted in gray in the wild-type (WT) sequence.

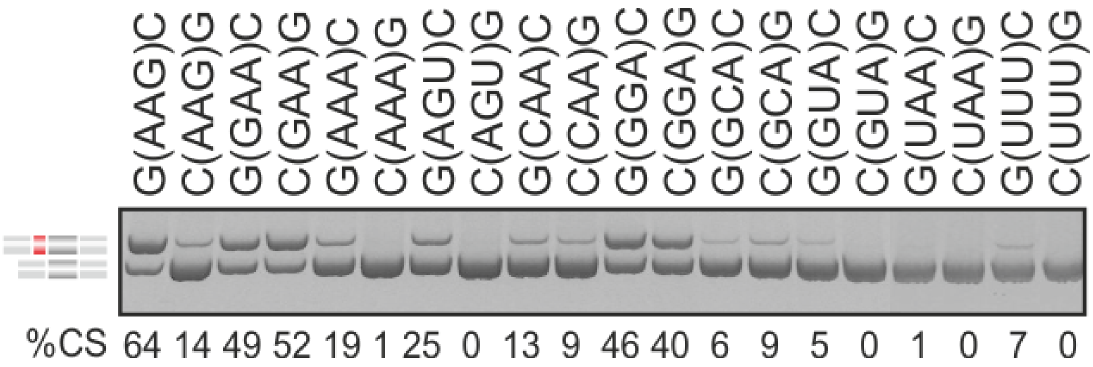

Figure S5
Triloop-closing base-pairs and MIR exon selection

Terminal triloops of the MIR hairpin are shown in parentheses in the context of GC and CG closing base pairs (top). RNA products are schematically shown to the left, percentage of cryptic splicing (%CS) at the bottom.

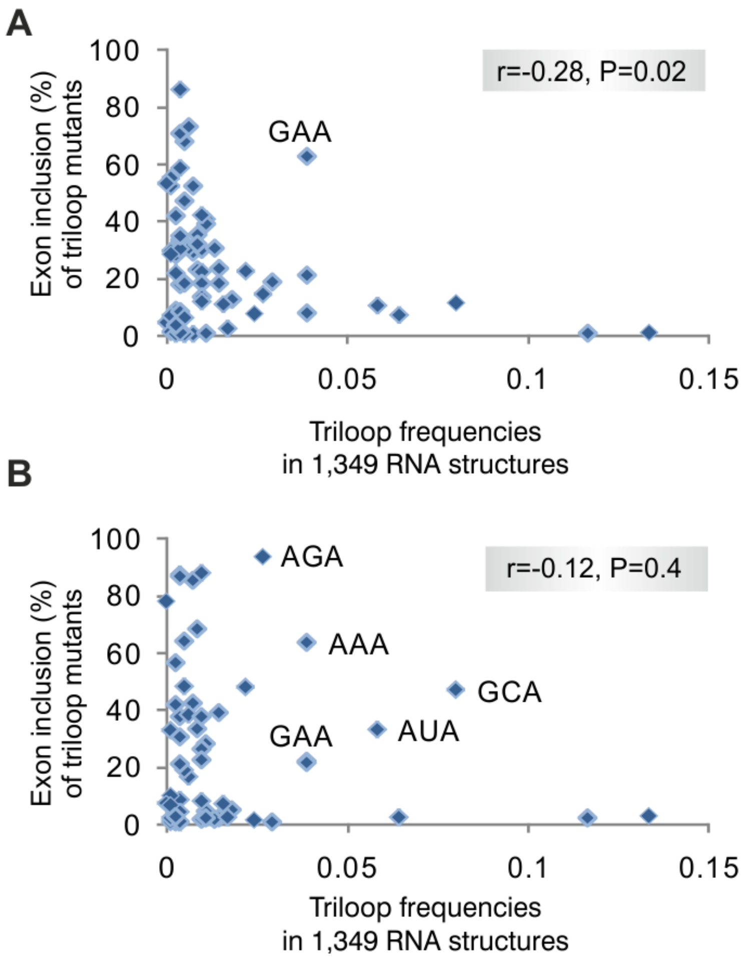

Figure S6
Trilooop frequencies in previously determined RNA secondary structures inversely correlate with MIR exon inclusion levels of matching terminal triloops in *FGB* transcripts

(A) terminal loop mutants. **(B)** internal triloop mutants. Exon inclusion levels are means of duplicate transfections into COS7 cells.

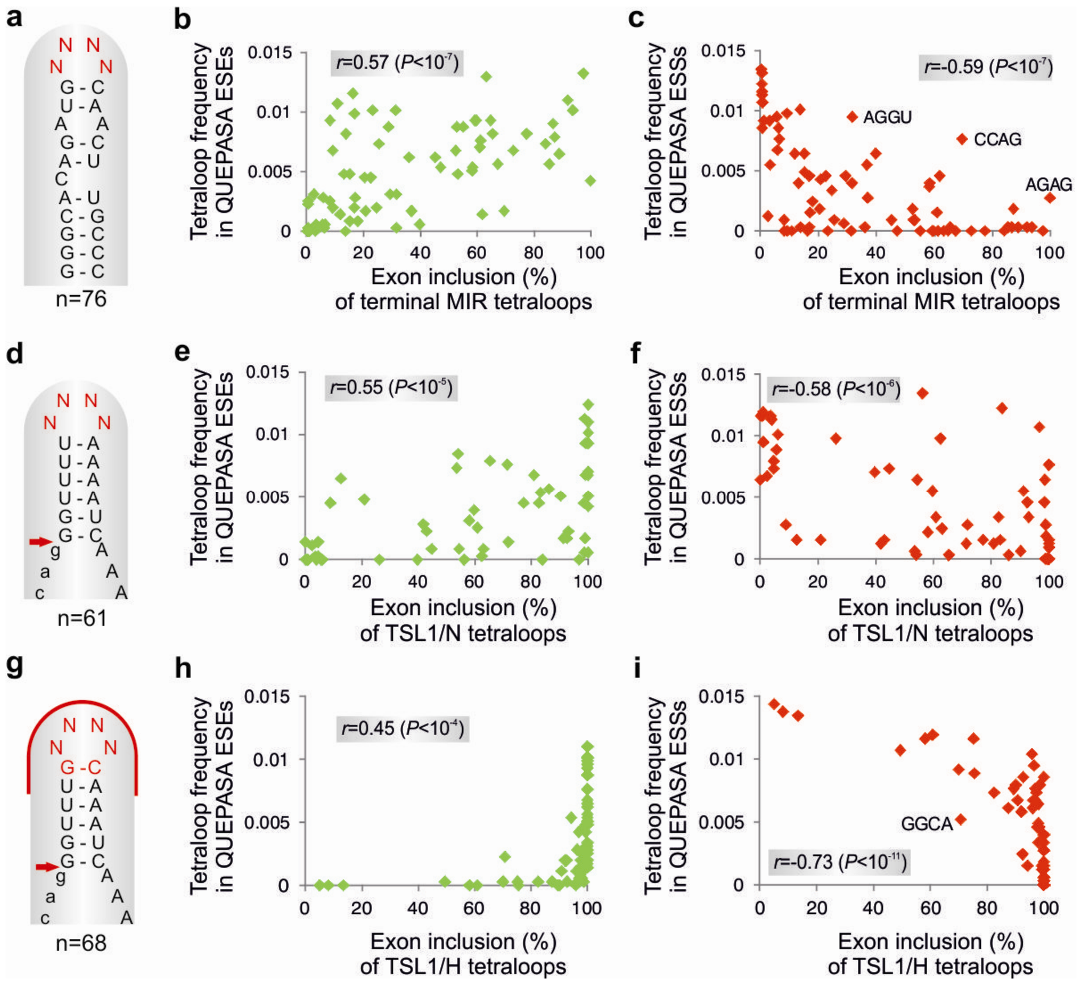

Figure S7
Tetranucleotide frequencies in enhancers and silencers correlate with exon inclusion levels of corresponding terminal tetraloops

(A-C) MIR hairpin in the *FGB* minigene. **(D-F)** TSL1/N hairpin in the *SMN2* minigene. **(G-I)** TSL1/H hairpin with mutated loop-closing base pairs**.** Mutated nucleotides are shown in red. The 3' splice site is indicated by a red arrow. Enhancers and silencers are shown as green and red symbols, respectively. Octamer sequence in TSL1/H identical to the MIR hairpin in *FGB* is denoted by a red curve in panel **G**.

A

ACUUCUAGCAAUACAGGAUUACAAUUAAGAGGACAAGAUCUGAAAAUCUCACAAACUAUAAAAUAAUAAAAGAGCAGAAUUUUAAGAUAAAAGAAACUGGUGGUAGGUAG

B

UUUUACAGAUGAGAAAACUGGGGCACAGAUAAAGCAACUUGCCCAAGGUCUCAUAGCUGUAAGUCA

Figure S8
Predicted optimal Tra2β binding sites in T and MIR exons

(A) T exon. **(B)** MIR exon (WT). Binding sites are shown in red, exons are highlighted in gray.

++

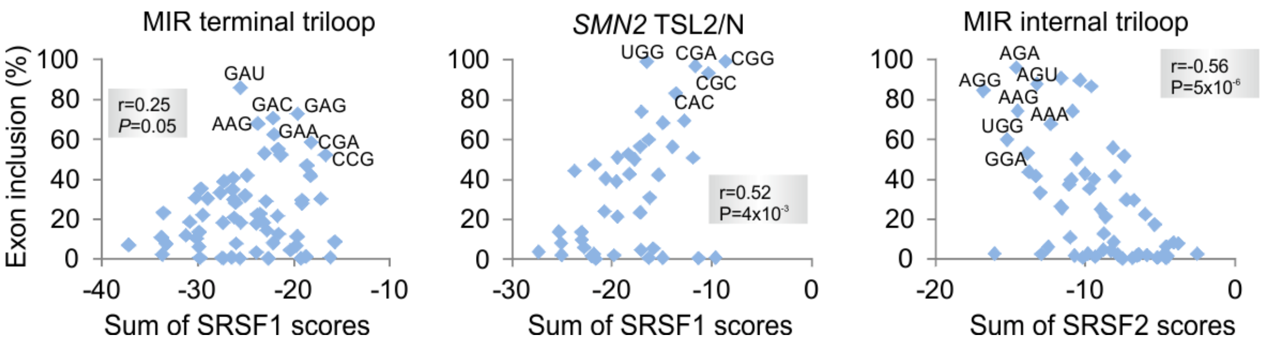

Figure S9
Correlation between exon inclusion and scores for predicted exonic splicing regulatory sequences of SR proteins

Scores were computed by ESEfinder (v. 3.0) [2] for each position of 13-mers that encompassed loops shown at the top. Triloops contributing most to positive or negative correlation are labelled.

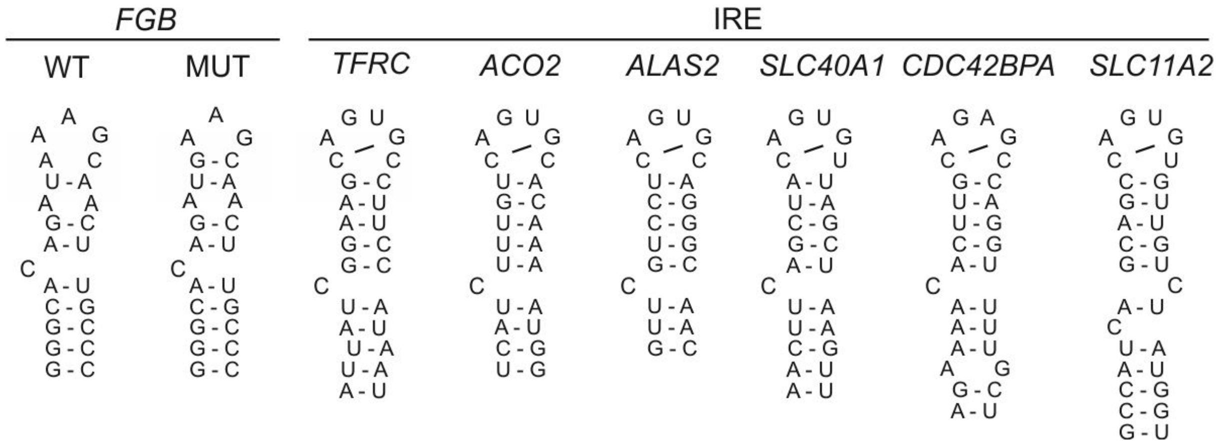

Figure S10
Similarities between *FGB* and IRE hairpins

IRE hairpins are shown for *TFRC* (transferrin receptor), *ACO2* (mitochondrial aconitase), *ALAS2* (erythroid aminolevulinic synthase), *SLC40A1* (ferroportin), *CDC42BPA* (CDC42-binding protein kinase α) and *SLC11A2* (divalent metal ion transporter). IRE secondary structure predictions are as published previously [3].

SUPPLEMENTARY TABLES

	Splicing enhancers					Splicing silencers						Neutral	
	RESCUE-ESEs	PESEs	EIEs	Trusted NI ESEs	ESEseqs (QUEPASA)	FAS-ESSs (hex2)	FAS-ESSs (hex3)	PESSs	IIEs	Trusted NI ESSs	ESSseqs (QUEPASA)	NEUTRAL QUEPASA	ESRs
Number of elements/ nucleotides	238/ 1,428	2060/ 16,480	1131/ 6,786	673/ 4,038	1,182/ 7,092	176/ 1,056	103/ 618	1019/ 8,152	708/ 4,248	386/ 2.316	1,090/ 6,540	1,824/ 10,944	285/ 1,710
Reference	4	5	6	7	8	9	9	5	6	7	8	8	10
Terminal MIR triloop (n=64, HEK293)	0.42 (*0.0005*)	0.46 (*0.0002*)	0.31 (*0.01*)	0.49 (*0.00004*)	**0.57 (*0.0000007*)**	-0.24 (*0.06*)	-0.23 (*0.06*)	-0.29 (*0.02*)	-0.19 (*0.16*)	-0.33 (*0.008*)	**-0.50 (*0.00003*)**	-0.08 (*NS*)	0.04 (*NS*)
Terminal MIR triloop (n=64, HepG2)	0.50 (*0.00003*)	0.48 (*0.00005*)	0.32 (*0.009*)	0.53 (*0.000008*)	**0.60 (*0.0000001*)**	-0.29 (*0.02*)	-0.28 (*0.02*)	-0.31 (*0.01*)	-0.21 (*0.09*)	-0.36 (*0.003*)	**-0.56 (*0.000002*)**	-0.02 (*NS*)	0.06 (*NS*)
Terminal MIR triloop (n=64, HeLa)	0.26 (*0.04*)	0.29 (*0.02*)	0.18 (*NS*)	0.33 (*0.008*)	**0.46 (*0.0001*)**	-0.17 (*NS*)	-0.17 ()*NS*	-0.25 (*0.05*)	-0.09 (*NS*)	-0.26 (*0.04*)	**-0.41 (*0.0007*)**	-0.04 (*NS*)	-0.03 (*NS*)
Terminal MIR triloop (n=64, COS7)	0.45 (*0.0002*)	0.47 (*0.00008*)	0.29 (*NS*)	0.50 (*0.00002*)	**0.60 (*0.0000002*)**	-0.30 (*0.01*)	-0.31 (*0.01*)	-0.32 (*0.009*)	-0.20 (*NS*)	-0.38 (*0.002*)	**-0.56 (*0.000001*)**	0.03 (*NS*)	0.07 (*NS*)
Terminal MIR tetraloop (n=76, COS7)	0.36 (*0.001*)	0.53 (*0.0000007*)	0.36 (*0.001*)	0.53 (*0.0000007*)	**0.57 (*0.00000008*)**	-0.39 (*0.0005*)	-0.37 (*0.0009*)	-0.34 (*0.003*)	-0.30 (*0.008*)	-0.44 (*0.00006*)	**-0.59 (0.00000002)**	-0.02 (*NS*)	0.10 (*NS*)
Internal MIR triloop (n=57, COS7)	**0.35 (0.007)**	0.22 (NS)	0.26 (0.05)	0.28 (0.03)	0.21 (NS)	-0.14 (NS)	-0.14 (NS)	-0.09 (NS)	-0.15 (NS)	-0.15 (NS)	-0.14 (NS)	-0.07 (NS)	-0.19 (NS)
FGB-SMN2 hybrid (n=62, HEK293)	0.51 (0.00002)	0.48 (0.00007)	0.38 (0.002)	0.51 (0.00002)	**0.57 (0.000001)**	-0.36 (0.004)	-0.36 (0.004)	-0.30 (0.02)	-0.33 (0.01)	-0.40 (0.001)	**-0.58 (0.0000007)**	-0.04 (NS)	-0.02 (NS)
FGB-F9 hybrid (n=36, COS7)	0.44 (0.007)	0.48 (0.003)	0.39 (0.02)	**0.52 (0.001)**	0.42 (0.01)	-0.28 (NS)	-0.27 (NS)	-0.18 (NS)	-0.22 (NS)	-0.28 (NS)	**-0.43 (0.009)**	0.03 (NS)	0.13 (NS)
FGB-L1CAM hybrid (r1) (n=61, COS7)	**0.43 (0.0005)**	0.30 (0.02)	0.28 (0.03)	0.37 (0.003)	0.32 (0.01)	-0.14 (NS)	-0.14 (NS)	-0.10 (NS)	-0.03 (NS)	-0.14 (NS)	**-0.30 (0.02)**	-0.04 (NS)	0.09 (NS)
FGB-L1CAM hybrid (r2) (n=43, COS7)	0.57 (0.00007)	0.55 (0.0001)	0.47 (0.001)	**0.60 (0.00002)**	0.49 (0.0008)	-0.50 (0.0006)	-0.53 (0.0003)	-0.24 (NS)	-0.24 (NS)	-0.46 (0.002)	**-0.64 (0.000004)**	0.42 (0.006)	0.36 (0.02)
Native SMN2 (TSL1/N) (n=60, HEK293; tetra)	0.09 (NS)	0.34 (0.008)	0.16 (NS)	0.29 (0.03)	**0.55 (0.000005)**	-0.22 (NS)	-0.22 (NS)	**-0.63 ($7x10^{-8}$)**	-0.25 (NS)	-0.54 (0.00001)	-0.58 (0.000001)	0.11 (NS)	0.25 (NS)
Native SMN2 (TSL2/N) (n=42, HEK 293; tri)	0.08 (NS)	0.21 (NS)	0.11 (NS)	0.22 (NS)	**0.44 (0.004)**	-0.29 (NS)	-0.30 (NS)	-0.45 (0.003)	-0.39 (0.01)	-0.47 (0.002)	**-0.55 (0.0002)**	0.19 (NS)	0.03 (NS)
Native SMN2 (TSL1/H) (n=68, HEK 293; tetra)	0.23 (NS)	0.25 (0.04)	0.37 (0.002)	0.30 (0.01)	**0.45 (0.0001)**	-0.65 ($2x10^{-9}$)	-0.68 (10^{-10})	-0.27 (0.03)	-0.06 (NS)	-0.56 ($8x10^{-7}$)	**-0.73 ($2x10^{-12}$)**	0.52 (0.000006)	0.24 (0.05)
Native SMN2 (TSL2/H) (n=42, HEK 293; tri)	0.25 (NS)	0.39 (0.01)	0.25 (NS)	0.40 (0.009)	**0.53 (0.0003)**	-0.31 (0.05)	-0.26 (NS)	-0.57 (0.00007)	-0.27 (NS)	-0.58 (0.00005)	**-0.60 (0.00003)**	0.17 (NS)	0.25 (NS)

Table S1
Correlation matrix for exon inclusion levels of tri-/tetra-loop mutants and corresponding tri-/tetra-nucleotide frequencies in splicing enhancers and silencers

Abbreviations of auxiliary splicing elements (top) are explained in the Materials and Methods section and Supplementary references. Constructs, hairpins, number of mutants (n) and transfected cell lines are shown in the first column. Pearson correlation coefficients are followed by *P* values in parentheses. The most significant silencer and enhancer *P* values in each table row are highlighted in red.

Internal triloop	Number of extra H bonds	Internal triloop	Number of extra H bonds
AAA	0	GAA	5
AAC	3	GAC	8
AAG	0	GAG	5
AAU	2	GAU	7
ACA	3	GCA	3
ACC	3	GCC	3
ACG	3	GCG	3
ACU	3	GCU	3
AGA	0	GGA	5
AGC	5	GGC	8
AGG	0	GGG	5
AGU	2	GGU	7
AUA	0	GUA	0
AUC	3	GUC	6
AUG	0	GUG	0
AUU	2	GUU	2
CAA	3	UAA	2
CAC	3	UAC	3
CAG	3	UAG	2
CAU	3	UAU	2
CCA	3	UCA	2
CCC	3	UCC	3
CCG	3	UCG	2
CCU	3	UCU	2
CGA	3	UGA	2
CGC	5	UGC	5
CGG	3	UGG	2
CGU	3	UGU	2
CUA	3	UUA	2
CUC	3	UUC	3
CUG	3	UUG	2
CUU	3	UUU	2

Table S2
Predicted number of hydrogen bonds between each internal triloop and the antiparallel strand of the MIR hairpin

Primer	Sequence (5'-3')
**Cloning of the *FGB* minigene**	
C-F (cloning, forward)	tat tat tgc caa gta ctg ttc
C-R (cloning, reverse)	aat gta act att cca aca ccc
S (sequencing)	tcg aag tgg aga gga cac
**Cloning of hybrid constructs**	
SMN2-FGB-F	ggg cac aga tga agc aac ttg ccc aaa tta agg agt aag tct
SMN2-FGB-R	ggg caa gtt gct tca tct gtg ccc ctt ttt gat ttt gtc taa aac
F9-FGB-F	ggg cac aga tga agc aac ttg ccc aag cag tat gtt ggt aag c
F9-FGB-R	ggg caa gtt gct tca tct gtg ccc caa aat tca gtc tat aaa
L1CAM(r1)-FGB-F1	ggg cac aga tga agc aac ttg ccc aag tgc cgt gct ggt caa g
L1CAM(r1)-FGB-R1	ggg caa gtt gct tca tct gtg ccc cag ctc agg gat tgc ctg
L1CAM(r2)-FGB-F2	ggg cac aga tga agc aac ttg ccc tcc gcg gat aca atg taa g
L1CAM(r2)-FGB-R2	ggg caa gtt gct tca tct gtg ccc cca ccg gcc gcc act tga
**Primers for triloop mutagenesis**	
M (*FGB*)	tgg ggc aca gat gnn nca act tgc cca a
SMN2-NNN	aat caa aaa ggg gca cag atg nnn caa ctt gcc caa att aag ga
F9-NNN	act gaa ttt tgg ggc aca gat gnn nca act tgc cca agc agt atg tt
L1CAM(*r1*)-NNN	aat ccc tga gct ggg gca cag atg nnn caa ctt gcc caa gtg ccg t
L1CAM(r2)-NNN	cgg ccg gtg ggg gca cag atg nnn caa ctt gcc ctc cgc gga t
**Cloning of in vitro splicing reporter**	
PY7-FGB-F-*Xho*I	att act cga gct tgt aag gca gat tca aa
PY7-FGB-R-*Bam*HI	ata ggg atc cga ctt aca gct atg aga cct
pCR-PY7F-*Nhe*I	att agc tag ctt gtc gag gag gac a
**Cloning of expression plasmids**	
Tra2β-F-*Bam*HI	att agg atc cat gag cga cag cgg cga gca
Tra2β-R-*Not*I	att agc ggc cgc tta ata gcg acg agg tga gt
Tra2α-F-*Bam*HI	att agg atc cat gag tga tgt gga gga aaa c
Tra2α-R-*Not*I	att agc ggc cgc ttc cgt tat caa tag cgt ctt
**Mutagenesis of expression plasmids**	
Tra2β-F163A	gag gat ttg ccg ctg tat att ttg aa
Tra2α-F163A	gag gat ttg ctg ctg tgt att ttg a
Tra2β-F193A	atc aga gtt gat gcc tct ata aca aaa
Tra2α-Y193A	gaa ttc ggg tgg atg ctt cta taa cca a
Tra2β-R190A	ggg cgt agg atc gca gtt gat ttc tct
Tra2α-R190A	ggt aga aga att gcg gtg gat tat tct
**Detection of spliced products in exogenous transcripts using RT-PCT**	
PL1	act cac tat agg gag acc
PL2	ggc tga tca gcg ggt tta
**Antisense oligonucleotides**	
SSO-AAG	agu ugc uuc auc u
SSO-GAU	agu uga ucc auc u
SSO-5'stem	auc ugu gcc cca g
SSO-3'stem	uug ggc aag uug

Table S3 Primers

Antisense oligoribonucleotide were 2'-*O*-methyl-modified at each sugar residue and uniformly labeled with phosphorothioates.

Triloop	Exon inclusion (%)	Tetraloop	Exon inclusion (%)
AAA	20.96	AGAG	99.91
AAC	40.65	GACG	97.80
AAG	67.99	GAUG	95.34
AAT	35.34	GAAG	92.89
ACA	22.47	GCCG	89.55
ACC	17.80	GGAG	89.50
ACG	52.29	GAGA	88.92
ACT	38.95	GAGC	88.04
AGA	14.48	ACUG	87.78
AGC	29.06	GCGA	79.27
AGG	4.52	ACCG	75.89
AGT	29.91	CCAG	74.00
ATA	10.46	UCUG	69.93
ATC	30.76	UCCG	69.41
ATG	33.29	CCUG	67.50
ATT	23.33	CGCG	67.20
CAA	12.67	GCUG	67.15
CAC	21.70	AACG	64.87
CAG	29.65	ACAG	64.72
CAT	32.00	GGGA	64.17
CCA	28.02	CGAG	62.77
CCC	0.46	GAGG	62.32
CCG	52.25	GAAA	61.13
CCT	0.49	GGAA	59.60
CGA	58.61	AUCG	58.27
CGC	41.85	GUCG	58.11
CGG	8.73	GUGA	57.26
CGT	55.20	CAAG	53.32
CTA	18.35	AGCG	47.83
CTC	0.47	UCAG	42.25
CTG	53.12	UGAG	37.43
CTT	30.48	CACG	37.38
GAA	62.53	GCAG	35.43
GAC	70.79	CGUG	33.45
GAG	72.92	AGGU	33.42
GAT	86.04	AAUG	32.88
GCA	11.39	AAGG	31.88
GCC	6.74	GCGG	30.45
GCG	30.31	AAAG	28.15
GCT	18.38	GCAA	26.94
GGA	46.98	UGCG	26.51
GGC	1.32	GUUG	25.73
GGG	0.92	ACGG	23.25
GGT	8.19	CUCU	22.81
GTA	7.91	AUCA	20.56
GTC	28.39	AUUG	20.46
GTG	22.85	GUGG	19.21
GTT	22.24	CUGG	18.90
TAA	0.75	GGGU	17.68
TAC	6.20	UUCG	17.66
TAG	0.46	CCGG	16.85
TAT	7.64	CCCG	16.54
TCA	18.33	AUGG	16.36
TCC	0.67	AGUG	16.35
TCG	42.05	AGCU	14.41
TCT	18.7	UCGG	13.12
TGA	34.89	UACG	10.14
TGC	0.98	CAUG	9.86
TGG	3.45	CCCC	9.54
TGT	13.59	CUCG	9.44
TTA	2.44	UGUG	7.75
TTC	10.95	UUUG	7.75
TTG	11.88	GGUG	7.65
TTT	7.15	CAGG	6.54
		UAUG	4.1
		CUUG	3.63
		GGCC	3.3
		GUAA	1.54
		AUAG	0.79
		UGGG	0.68
		UAAG	0.66
		GUAG	0.65
		CUAG	0.55
		GGGG	0.51
		AGGG	0.46
		UAGG	0.12

Table S4 Mean exon inclusion levels of 64 terminal triloop and 76 tetraloop *FGB* mutants transfected into COS7 cells.

SUPPLEMENTARY REFERENCES

1. Corvelo A, Hallegger M, Smith CW, Eyras E. Genome-wide association between branch point properties and alternative splicing. PLoS Comput Biol 2010; 6:e1001016.
2. Cartegni L, Wang J, Zhu Z, Zhang MQ, Krainer AR. ESEfinder: a web resource to identify exonic splicing enhancers. Nucleic Acids Res 2003; 31:3568-71.
3. Piccinelli P, Samuelsson T. Evolution of the iron-responsive element. RNA 2007; 13:952-66.
4. Fairbrother WG, Yeo GW, Yeh R, Goldstein P, Mawson M, Sharp PA, et al. RESCUE-ESE identifies candidate exonic splicing enhancers in vertebrate exons. Nucleic Acids Res 2004; 32:W187-90.
5. Zhang XH, Chasin LA. Computational definition of sequence motifs governing constitutive exon splicing. Genes Dev 2004; 18:1241-50.
6. Zhang C, Li WH, Krainer AR, Zhang MQ. RNA landscape of evolution for optimal exon and intron discrimination. Proc Natl Acad Sci USA 2008; 105:5797-802.
7. Stadler MB, Shomron N, Yeo GW, Schneider A, Xiao X, Burge CB. Inference of splicing regulatory activities by sequence neighborhood analysis. PLoS Genet 2006; 2:e191.
8. Ke S, Shang S, Kalachikov SM, Morozova I, Yu L, Russo JJ, et al. Quantitative evaluation of all hexamers as exonic splicing elements. Genome Res 2011; 21:doi10.1101/gr.119628.110.
9. Wang Z, Rolish ME, Yeo G, Tung V, Mawson M, Burge CB. Systematic identification and analysis of exonic splicing silencers. Cell 2004; 119:831-45.
10. Goren A, Ram O, Amit M, Keren H, Lev-Maor G, Vig I, et al. Comparative analysis identifies exonic splicing regulatory sequences. The complex definition of enhancers and silencers. Mol Cell 2006; 22:769-81.
